# Telomere Disruption Results in Non-Random Formation of *De Novo* Dicentric Chromosomes Involving Acrocentric Human Chromosomes

**DOI:** 10.1371/journal.pgen.1001061

**Published:** 2010-08-12

**Authors:** Kaitlin M. Stimpson, Ihn Young Song, Anna Jauch, Heidi Holtgreve-Grez, Karen E. Hayden, Joanna M. Bridger, Beth A. Sullivan

**Affiliations:** 1 Duke Institute for Genome Sciences and Policy, Duke University, Durham, North Carolina, United States of America; 2 Department of Genetics and Genomics, Boston University School of Medicine, Boston, Massachusetts, United States of America; 3 Institute of Human Genetics, University Hospital Heidelberg, Heidelberg, Germany; 4 Laboratory of Nuclear and Genomic Health, Centre for Cell and Chromosome Biology, Division of Biosciences, Brunel University, Uxbridge, United Kingdom; 5 Department of Molecular Genetics and Microbiology, Duke University Medical Center, Durham, North Carolina, United States of America; The University of North Carolina at Chapel Hill, United States of America

## Abstract

Genome rearrangement often produces chromosomes with two centromeres (dicentrics) that are inherently unstable because of bridge formation and breakage during cell division. However, mammalian dicentrics, and particularly those in humans, can be quite stable, usually because one centromere is functionally silenced. Molecular mechanisms of centromere inactivation are poorly understood since there are few systems to experimentally create dicentric human chromosomes. Here, we describe a human cell culture model that enriches for *de novo* dicentrics. We demonstrate that transient disruption of human telomere structure non-randomly produces dicentric fusions involving acrocentric chromosomes. The induced dicentrics vary in structure near fusion breakpoints and like naturally-occurring dicentrics, exhibit various inter-centromeric distances. Many functional dicentrics persist for months after formation. Even those with distantly spaced centromeres remain functionally dicentric for 20 cell generations. Other dicentrics within the population reflect centromere inactivation. In some cases, centromere inactivation occurs by an apparently epigenetic mechanism. In other dicentrics, the size of the α-satellite DNA array associated with CENP-A is reduced compared to the same array before dicentric formation. Extra-chromosomal fragments that contained CENP-A often appear in the same cells as dicentrics. Some of these fragments are derived from the same α-satellite DNA array as inactivated centromeres. Our results indicate that dicentric human chromosomes undergo alternative fates after formation. Many retain two active centromeres and are stable through multiple cell divisions. Others undergo centromere inactivation. This event occurs within a broad temporal window and can involve deletion of chromatin that marks the locus as a site for CENP-A maintenance/replenishment.

## Author Summary

Endogenous human centromeres are defined by large arrays of α-satellite DNA. A portion of each α-satellite array is assembled into CENP-A chromatin, the structural and functional platform for kinetochore formation. Most chromosomes are monocentric, meaning they have a single centromere. However, genome rearrangement can produce chromosomes with two centromeres (dicentrics). In most organisms, dicentrics typically break during cell division; however, dicentric human chromosomes can be stable in mitosis and meiosis. This stability reflects centromere inactivation, a poorly understood phenomenon in which one centromere is functionally silenced. To explore molecular and genomic events that occur at the time of dicentric formation, we describe a cell-based system to create dicentric human chromosomes and monitor their behavior after formation. Such dicentrics can experience several fates, including centromere inactivation, breakage, or maintaining two functional centromeres. Unexpectedly, we also find that dicentrics with large (>20Mb) inter-centromeric distances are stable through at least 20 cell divisions. Our results highlight similarities and differences in dicentric behavior between humans and model organisms, and they provide evidence for one mechanism of centromere inactivation by centromeric deletion in some dicentrics. The ability to create dicentric human chromosomes provides a system to test other mechanisms of centromere disassembly and dicentric chromosome stability.

## Introduction

Chromosome inheritance requires essential chromosomal loci, namely centromeres, telomeres and origins of replication. Origins ensure precise copying of the entire genome, telomeres protect chromosome termini from degradation and deletion, and centromeres partition the copied genome to daughter cells. Defects in any of these functions lead to genome instability, rearrangement, and aneuploidy. Chromosome abnormalities are major factors in disease, reproductive failure, miscarriage and infertility. In addition, genome rearrangements (deletions, duplications, translocations, insertions, inversions) are a hallmark of many cancers [1]. The vast number of recurrent and non-recurrent cancer-related chromosome rearrangements highlights the scope of human genome instability (http://cgap.nci.nih.gov/Chromosomes/Mitelman) [2], [3]. Constitutive chromosome abnormalities also underlie congenital human diseases. Notwithstanding the frequency of these abnormalities, their origin and behavior at the time of formation are less clear, and can usually be inferred only from patient samples that are studied long after the rearrangements have occurred.

In humans, the most common structural chromosome rearrangement is the Robertsonian translocation (ROB) [4]. First described in insects [5], ROBs are formed by fusion at the centromere region between two acrocentric chromosomes. The term “acrocentric” refers to a chromosome in which the centromere is located very near one end, and the short arm may be difficult to observe cytologically. Humans have five pairs of acrocentric chromosomes, *Homo sapiens* chromosome (HSA) 13, HSA14, HSA15, HSA21 and HSA22. All acrocentric short arms contain homologous, but compositionally heterogeneous blocks of repetitive sequences that span the estimated 10–15Mb between the telomere and the α-satellite DNA of the centromere. These repeats include multiple copies of the ribosomal genes (rDNA) composed of subunits of 18S, 5.8S and 28S rDNA and an intergenic spacer [6]. The rDNA clusters appear as nucleolar organizing regions (NORs) around which the nucleolus is formed. Tandemly repeated rDNA units are flanked by multiple subfamilies of ß-satellite DNA [7], [8]. In addition, several different subfamilies of satellite III DNA are located between proximal ß-satellite arrays and the α-satellite DNA of the centromere [7], [9]–[11]. ROBs in humans are rarely formed by breakage within the centromere. Most are actually short arm fusions, and breaks occur within satellite III DNA [12]–[16]. Consequently, >90% of patient-derived ROBs have two centromeres and are structurally dicentric. In humans, rob(13;14) and rob(14;21) account for approximately 85% of all ROBs, suggesting that the participation of the five human acrocentric chromosomes in ROBs is non-random [12], [17], [18]. Genomic organization or chromosome-specific interactions may favor the formation of these particular ROBs. Alternatively, the prevalence of rob(13;14) and rob(14;21) may simply reflect a population bias for the most viable developmental outcomes.

The prevailing view of dicentric behavior, first described by Barbara McClintock in the late 1930s, is that they are inherently unstable, often going through successive rounds of anaphase bridging and breakage [19]–[22]. However, dicentric ROBs in humans can be unusually stable and are often inherited through meiosis. Their stability has been attributed to centromere inactivation, a process by which one centromere is functionally silenced [23]–[26]. Testing mechanisms of centromere inactivation has proven difficult because there are few experimental systems to produce *de novo* dicentric human chromosomes. Much more progress has been made in understanding normal centromere structure and function. Our view of inactive centromeres is largely based on comparisons between active and inactive centromeres. The centromere is a complex chromosomal locus where the kinetochore is formed and microtubules attach during cell division. A major component of functional centromeres is CENP-A (Centromere Protein A), a histone H3 variant that replaces canonical H3 to create unique centromeric nucleosomes [27], [28]. CENP-A marks centromeres physically by assembling into largely homotypic nucleosomes (two copies of CENP-A) that have a more rigid conformation than H3-containing nucleosomes [29], [30]. Centromeric chromatin is arranged as multiple subunits of CENP-A nucleosomes periodically interspersed with subunits of H3 nucleosomes that are dimethylated at lysine 4 [31], [32]. It is thought that the physically distinct nucleosomes and long-range chromatin organization together make a platform upon which the kinetochore is formed and to which additional centromere and kinetochore proteins are recruited [33]–[35]. In genomic terms, multi-megabase regions of repetitive α-satellite that are concentrated at the primary constriction define the human centromere locus [36], [37]. However, CENP-A and other centromere/kinetochore proteins sequentially assemble on only a portion of the α-satellite array [38]–[40].

Inactive centromeres of dicentric chromosomes lack key centromere and kinetochore proteins, such as CENP-A, CENP-C, and CENP-E [25], [26]. At metaphase, they do not have a defined primary constriction and morphologically resemble chromosome arms. Thus, centromere inactivation is predicted to involve exclusion of centromere proteins and chromatin remodeling so that the two centromeres on the dicentric are functionally distinct. Some inactive centromeres are underacetylated and heterochromatic, although it is not clear if these features correlate with terminal states of inactivation [41], [42] or are representative of all inactive centromeres.

Patient-derived dicentric chromosomes are discovered by chance, and probably represent the most stable dicentric chromosomes. The events responsible for centromeric silencing must have occurred long before ascertainment of the chromosome rearrangement. This assumption is supported by studies in model organisms showing that most engineered dicentric chromosomes in yeast, plants, and Drosophila are unstable [20], [22], [43]. A system in which human dicentrics could be created reproducibly and studied immediately at the time of formation would provide valuable insight into mechanisms of dicentric behavior and centromere inactivation and allow for comparisons to studies in model organisms. Here, we describe formation of *de novo* dicentric human chromosomes *in vitro* using transient expression of a mutant version of telomere protein TRF2 that disrupts telomere function [44]. Chromosome fusions that result from telomere dysfunction are non-random, with the majority of induced fusions occurring between acrocentric chromosomes that represent ∼20% of the human karyotype. Our studies suggest that one mechanism of centromere inactivation involves deletion of a fraction of the multi-megabase array of α-satellite DNA upon which CENP-A chromatin is assembled.

## Results

**Figure 1:**
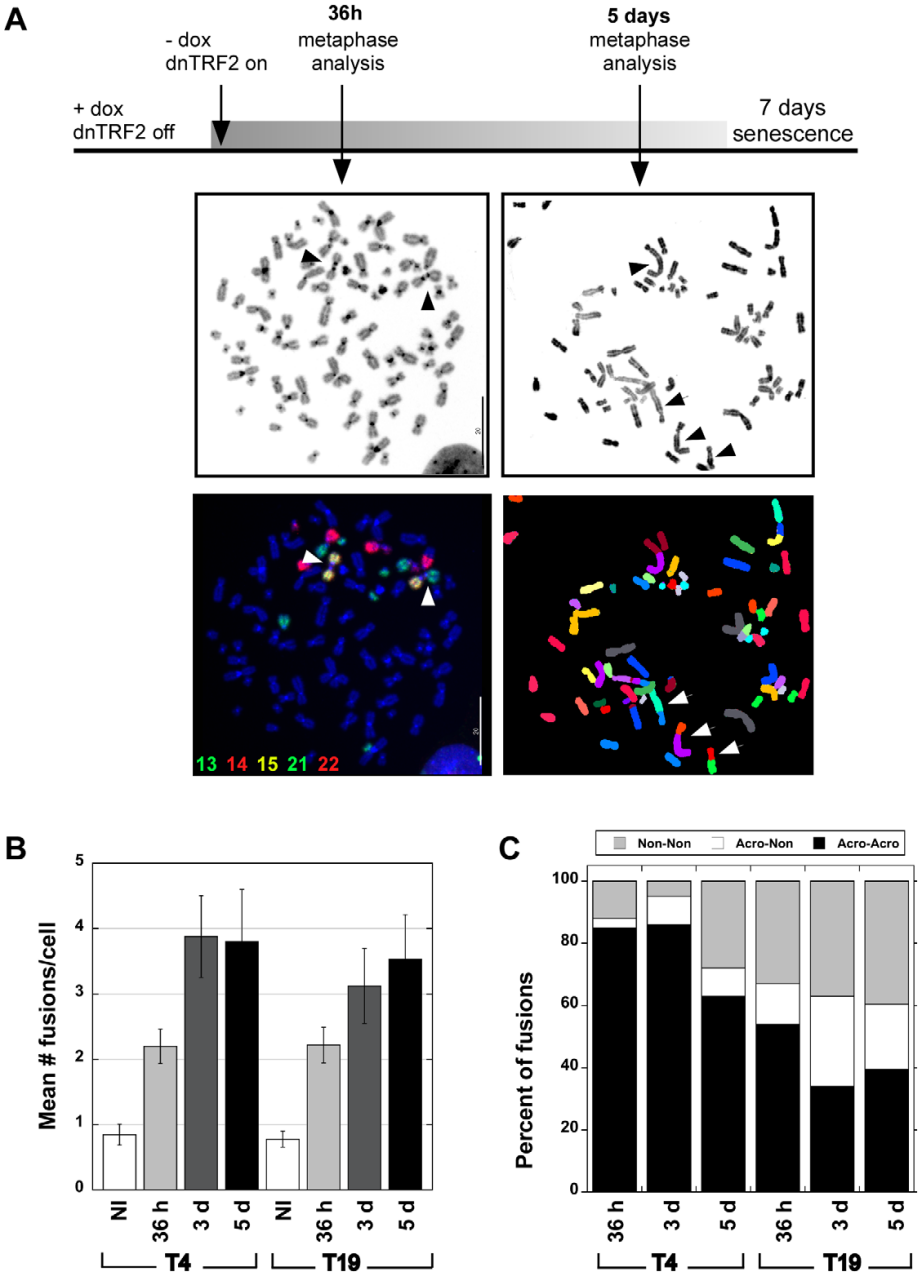
Human dicentric chromosomes are formed after transient TRF2^ΔBΔM^ (dnTRF2) expression. (A) Scheme for generating *de novo* dicentrics in HTC75 fibrosarcoma cells using inducible expression of mutant TRF2 (TRF2^ΔBΔM^). Short-term induction (36 hour) of dnTRF2 produced primarily dicentric chromosomes (arrowheads). Extended expression of dnTRF2 (5 days) resulted in multi-chromosome/multi-centric fusions (arrow). Chromosome fusions were identified using FISH with chromosome-specific painting probes and/or M-FISH. The gray-scale panel shows the DAPI-stained chromosomes from the same FISH image located below. The DAPI image was inverted (from black to white background) to reveal banding patterns on chromosomes. Scale bars = 20 µm. (B) Transient dnTRF2 expression generated ∼2 fusions per cell in two independently induced HTC75 clones, T4 and T19. Over 20 metaphases were analyzed for each time point (NI = not induced). (C) After short-term (36 hour) expression of dnTRF2 in independent inductions of T4 and T19, over 80% of fusions involved acrocentric chromosomes (black bars). As dnTRF2 expression extended to 3 days (3d) and 5 days (5d), the number of non-acrocentric fusions contributed to a greater proportion of the total fusions.

To produce *de novo* dicentric human chromosomes in a consistent and controlled manner, we modified an experimental system previously used to study telomere dynamics [44]. In this assay, expression of a tetracycline/doxycycline-responsive dominant-negative truncation mutant of the telomere protein TRF2 (TRF2^ΔBΔM^, hereafter called dnTRF2) sequesters endogenous TRF2 away from chromosome ends. It was established that prolonged induction (5–7 days) of dnTRF2 led to multi-chromosome end-to-end fusions and cell senescence [44]–[46]. However, we reasoned that transient dnTRF2 expression would avoid both senescence and complex chromosome fusions. The optimal induction time would produce one or only a few dicentric chromosomes per cell. We performed multiple independent inductions of dnTRF2 expression in two cell lines, HTC75_T19_ (T19) and HTC75_T4_ (T4), derivatives of parental HT1080 fibrosarcoma cells that are hypertetraploid. Cells were passaged in the absence of doxycyline (dox) for 24–120 hours (Figure 1A) to allow dnTRF2 expression. At each induction period (36 hours, 3 days and 5 days), metaphase chromosomes were isolated, and the number of chromosome fusions determined using FISH (Figure 1A). In uninduced T4 or T19 (control) lines, the background level of fusions was ∼0.8/cell (Figure 1B). This is an average estimate across the population, since over 60% of the uninduced cells contained no fusions at all. The presence of fusions in some cells was probably due to incomplete silencing of dnTRF2 expression, despite the presence of dox. This conclusion was supported by the absence of fusions in uninduced cells with increasing dox concentrations (data not shown). Additionally, HT1080 cells lacking the dnTRF2 construct exhibited no chromosomal fusions.

### Non-random chromosomal interactions: prevalence of acrocentric associations

As expected, long inductions of dnTRF2 (3 or 5 days) resulted in 3–15 fusions per cell (Figure 1A and 1B) that were often complex, multi-chromosome arrangements (Figure 1A, Figure S1A and S1B). Induction of dnTRF2 for 30–45 hours produced fewer dicentric chromosomes per cell, after which cells proliferated indefinitely when continuously grown in dox+ media. Cells from independent inductions were analyzed using FISH with chromosome-specific painting probes and/or M-FISH (multiplex fluorescence in situ hybridization). Less than 30% of uninduced cells contained a fusion, but in 36-hour inductions, >60% of cells contained two or more fusions. The number of induced cells that lacked fusions entirely was less than 15%. Expression of dnTRF2 expression for 36 hours produced more dicentric fusions in a greater number of cells and an increased number of dicentric fusions per cell compared to uninduced control/parental cells. Consequently, we focused our studies on the short-term, reversible inductions.

**Figure 2:**
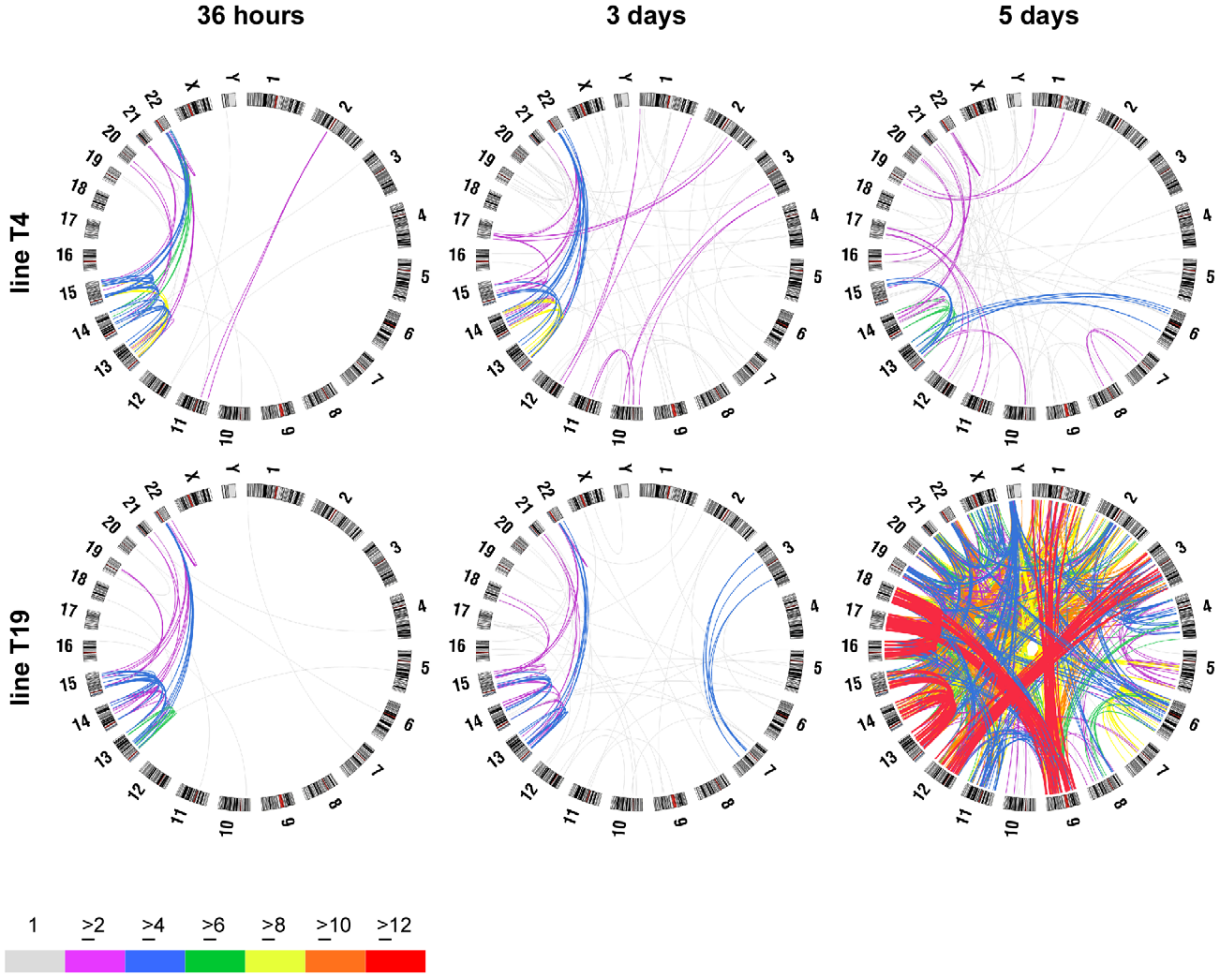
Circular maps illustrating chromosome fusions that occur after short- and long-term expression of dnTRF2. FISH was used to determine the frequency of specific chromosome fusions. Over 1000 fusions were scored in these analyses (≥25 metaphase/timepoint/cell line). Circle plots were generated using Circos (http://mkweb.bcgsc.ca/circos/) to visualize chromosome fusions over time after dnTRF2 expression. Chromosomes are shown along the outside of the circles. The lines are intended to represent fusions between specific chromosomes but do not designate precise breakpoints; line colors represent numbers of fusions (see color key). Acrocentric chromosome fusions represented the major fraction of dicentrics in short inductions of subclones T4 (top row) and T19 (bottom row). At 36 hours, most fusions occurred between acrocentrics. After 3- or 5-day dnTRF2 inductions, the percentage of non-acrocentric fusions increased. This is evident in the 5-day induction of T19 in which acrocentric interactions predominate (red lines), but HSA1-HSA9, HSA3-HSA12 and HSA9-HSA18 fusions also occurred frequently.

Chromosome fusions occurred non-randomly. In two independently induced lines T4 and T19, ∼80% of fusions involved acrocentrics (Figure 1C, Figure S1C). Most acrocentric fusions (138/174) involved two acrocentric chromosomes joined at the short (p) arms (Figure 1A, left panels), although we did observe p-q (short arm-long arm) and q-q fusions (Tables S1 and S2). This result was extremely statistically significant (χ^2^ test, *p*<0.0001) when compared to the expected proportion of random acrocentric interactions (4.6%). The remaining fusions involved either two non-acrocentric chromosomes, or a non-acrocentric and an acrocentric. It was clear from these experiments that interactions among the acrocentric chromosomes occurred quickly and frequently. When dnTRF2 was expressed for 3 or 5 days, the number of fusions per cell increased even more dramatically (Figure 1B, Figure 2), and this group included more non-acrocentric chromosome fusions (Figure 1C, Figure 2). Complex rearrangements (i.e. chains of 3–7 chromosomes), ring chromosomes, chromatid fusions, deleted chromosomes, and chromosome fragments were all observed (Figure S1; Tables S1 and S2). Importantly, even in longer inductions when more fusions occurred per cell, acrocentric-acrocentric fusions did not decrease. They consistently represented the largest proportion of all chromosome interactions in the cell population (Figure 1C, Figure S1C).

ROBs are the most prevalent chromosome translocation in humans. Acrocentric fusions (i.e. induced ROBs, hereafter referred to as iROBs) were also the most frequent in the inducible assay. In humans, rob(13;14) and rob(14;21) account for 85% of all ROBs [18]. However, irob(13;14) and irob(14;21) corresponded to only 16% (97/605) of all iROBs, suggesting that in our experimental system, all acrocentrics interact or more fusions are retained without potential selection bias (Figure S2A and S2B). The types and frequencies of iROBs differed over time, such that certain acrocentric associations occurred sooner than others (Figure S2A and S2B). Those involving the larger acrocentric chromosomes (HSA13, HSA14, and HSA15) occurred at similar frequencies in both short and long inductions, with the most prevalent iROB being irob(13;15) (16%), followed by irob(13;14) (13%). Fusions involving HSA21 and HSA22 were more common after 5-day inductions, with the exception of irob(15;22). Overall, iROBs occur non-randomly when telomeres are destabilized by dnTRF2. Some types of iROBs formed first, suggesting that some acrocentrics may be more predisposed to interact with each other or are more sensitive to telomere disruption.

### Induced fusions and the relationship to chromosomal proximity

Spatial location of nuclear chromosomal territories (CTs) within the nucleus influences chromosome interactions and translocation partners, particularly among non-random constitutive genomic exchanges and acquired translocations in cancers [3], [4], [47]–[49]. In humans, t(11;22) is the most prevalent constitutive non-Robertsonian translocation, while t(8;14), t(8;22), t(9;22) and t(15;17) are common acquired rearrangements in cancers [3], [50]. In our assay, the common constitutive and acquired rearrangements occurred at significantly lower rates than predicted by chance (Table S3). Interactions between chromosomes distantly located (HSA18-HSA19 or HSAX-HSA22) also did not exceed random fusion rates (*p* = 0.3) (Figure 2 and Figure S3, Tables S1, S2, and S3; Text S1), implying that the telomeres of these chromosomes were not closely located. Conversely, other fusions involving HSA1, HSA9, HSA17 and HSA18 occurred more frequently (Tables S1, S2, and S3). Two-dimensional analysis of CTs using chromosomal painting probes showed that HSA1, HSA17 and HSA18 were all peripherally located (Figure S3). Previous studies have shown that these same CTs intermingle [51], so presumably their telomeres reside near one another. We conclude that spatial characteristics of chromosomes contribute to non-random fusion that may be accentuated by destabilized telomeres.

### Not all induced dicentrics are telomere–telomere fusions

**Figure 3:**
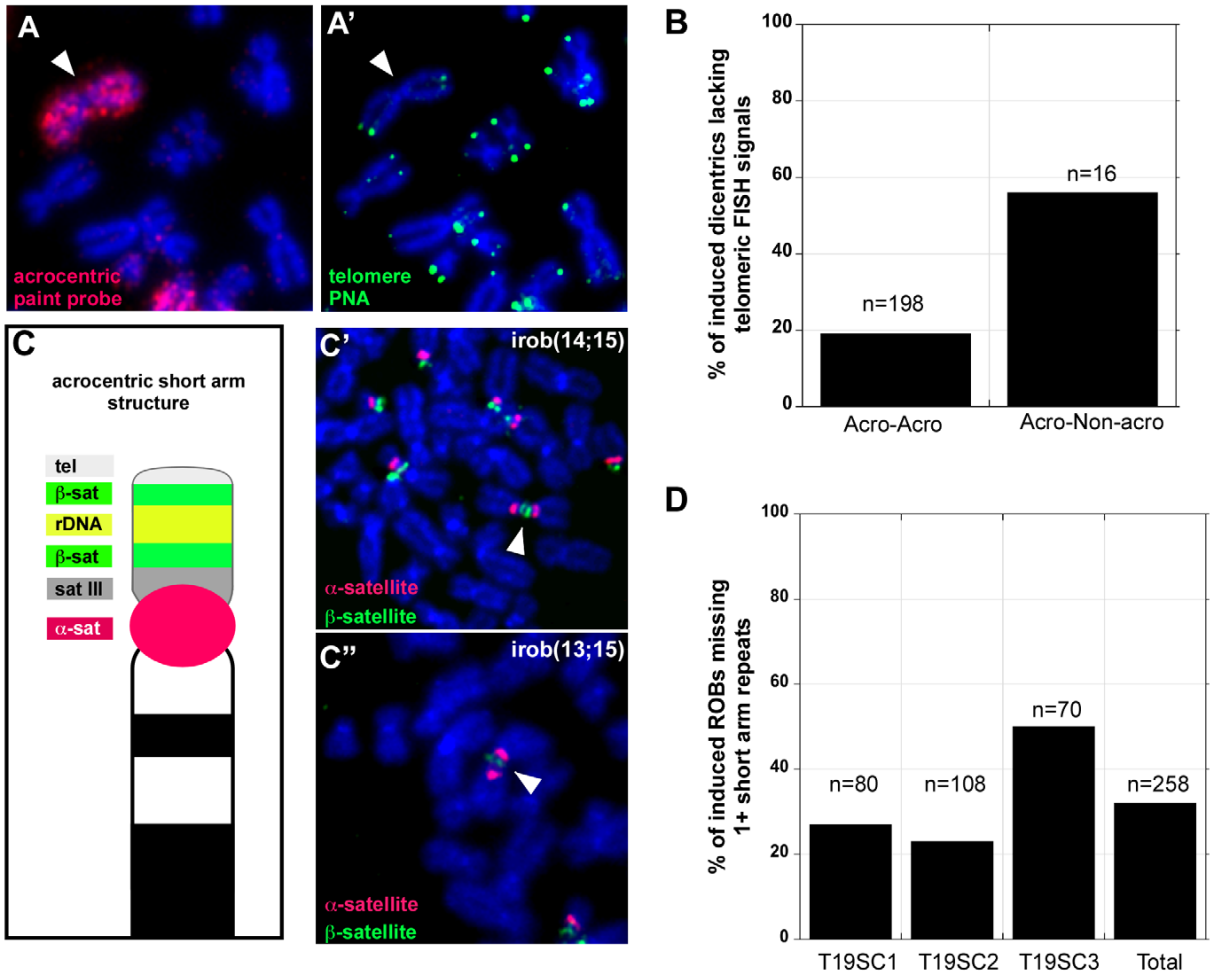
Heterogeneity in iROB structure: only half of induced dicentrics are true telomere-telomere fusions. (A, A′) FISH with PNA-telomere (green) and acrocentric painting (red) probes illustrated that an irob(14;14) lacked detectable telomeric repeats at the short arm fusion point. (B) Almost 20% of iROBs, and >50% of non-acrocentric-acrocentric fusions lacked telomeric FISH signals at the fusion breakpoints. (C) Schematic of acrocentric genomic organization. Multiple satellite repeats are located on all of the short arms of each acrocentric. (C′) FISH with acrocentric short arm specific probes revealed that an irob(14;15) retained distal ß-satellite array (green), implying that all repeats between the centromere (α-satellite, red) and the distal ß-satellite array were present on the iROB. (C″) Conversely, an irob(13;15) retained only a small amount of proximal ß-satellite (green) on only one of the acrocentrics, indicating heterogeneity in molecular structure of iROBs. (D) In three induced T19 subclones, FISH was used to assess the presence of acrocentric sequences on individual ROBs. Between 20% and 50% of iROBs lacked one or more short arm repeats.

Clearly, this experimental system produced massively heterogeneous populations of chromosomal fusions, opening the possibility to study many aspects of dicentric behavior. Although many non-acrocentric interactions were observed, the number of acrocentric fusions/iROBs still surpassed all others. Thus, as a first step, we concentrated our efforts on this subset of predominant chromosome fusions. Considering the biological basis of the dnTRF2 assay, we expected that most induced dicentrics were telomere-telomere fusions [44], [46], [52]. However, ∼20% (38/198) of iROBs lacked cytologically detectable telomeres at the point of fusion (Figure 3A, 3A′, and 3B). The remaining iROBs had variable numbers of telomere signals (1–3) (Figure S2C). Most acrocentric/non-acrocentric fusions (60%) also lacked detectable telomeric sequences at breakpoint junctions (Figure 3B). We next tested for sequences proximal to the telomere. In three independent clones, 32% (84/259) of iROBs lacked one or more short arm repeats, including rDNA sequences and pß4 ß-satellite arrays (Figure 3C, 3C′, 3C″, and 3D). In subclone T19SC3, one or more of the short arm sequences were missing in half of the iROBs (36/71). Whether a particular type of iROB retained all or some repeats appeared non-random (Figure S2D). Most irob(13;13) and irob(13;22) maintained all repeats (90% and 92%, respectively). Overall, though, one or both acrocentric chromosomes in the iROBs lacked one or more short arm repeats (Figure S2D). Consequently, iROBs produced in this assay exhibited variable inter-centromeric distances, conservatively estimated to range from 2Mb to 20Mb. From these experiments, we conclude that dnTRF2 not only affects telomere function, but impacts the stability of acrocentric short arm DNA located several megabases away from the telomere.

### Damage within acrocentric short arms coincides with iROB formation

Unprotected chromosomal ends are recognized as double-strand breaks, triggering a DNA damage response and recruitment of histone variant H2A.X phosphorylated at serine 139 (γH2AX) and DNA repair proteins [46], [53], [54]. Since many iROBs lacked telomere and short arm sequences, we used immunocytochemistry to determine if DNA breaks occurred in the acrocentric short arm at the time of dicentric formation. We observed the expected increase in γH2AX foci in interphase nuclei after expressing dnTRF2 for 36–48 hours (Figure S4A, S4B, S4C, S4A′, S4B′, and S4C′). Significantly more foci were associated with telomeres compared to control cells (Figure S4A, S4A′, and S4D; *p*<0.0001). The number of γH2AX foci that coincided with ß-satellite DNA was also significantly higher in dnTRF2 cells (Figure S4B, S4B′ and S4D; *p*<0.001). Although DNA damage was more prevalent throughout the nucleus after dnTRF2 expression, we did not observe increased γH2AX at control genomic sites (non-acrocentric satellite repeats or two euchromatic loci) (Figure S4C, S4C′, and S4D), suggesting that the increased damage was specific to telomeres and acrocentric short arms. Telomere detection and breakpoint analyses were performed at the time of iROB formation (36–48 hours), before cells had progressed through additional cell cycles. Thus, it seems unlikely that ongoing dicentric instability via breakage and re-fusion cycles was responsible for heterogeneous iROB structure. We conclude that iROBs in dnTRF2 cells were formed by telomere-telomere fusion or breakage within acrocentric short arm repeats.

### Acrocentric short arm and nucleolar organization are affected by dnTRF2 expression

**Figure 4:**
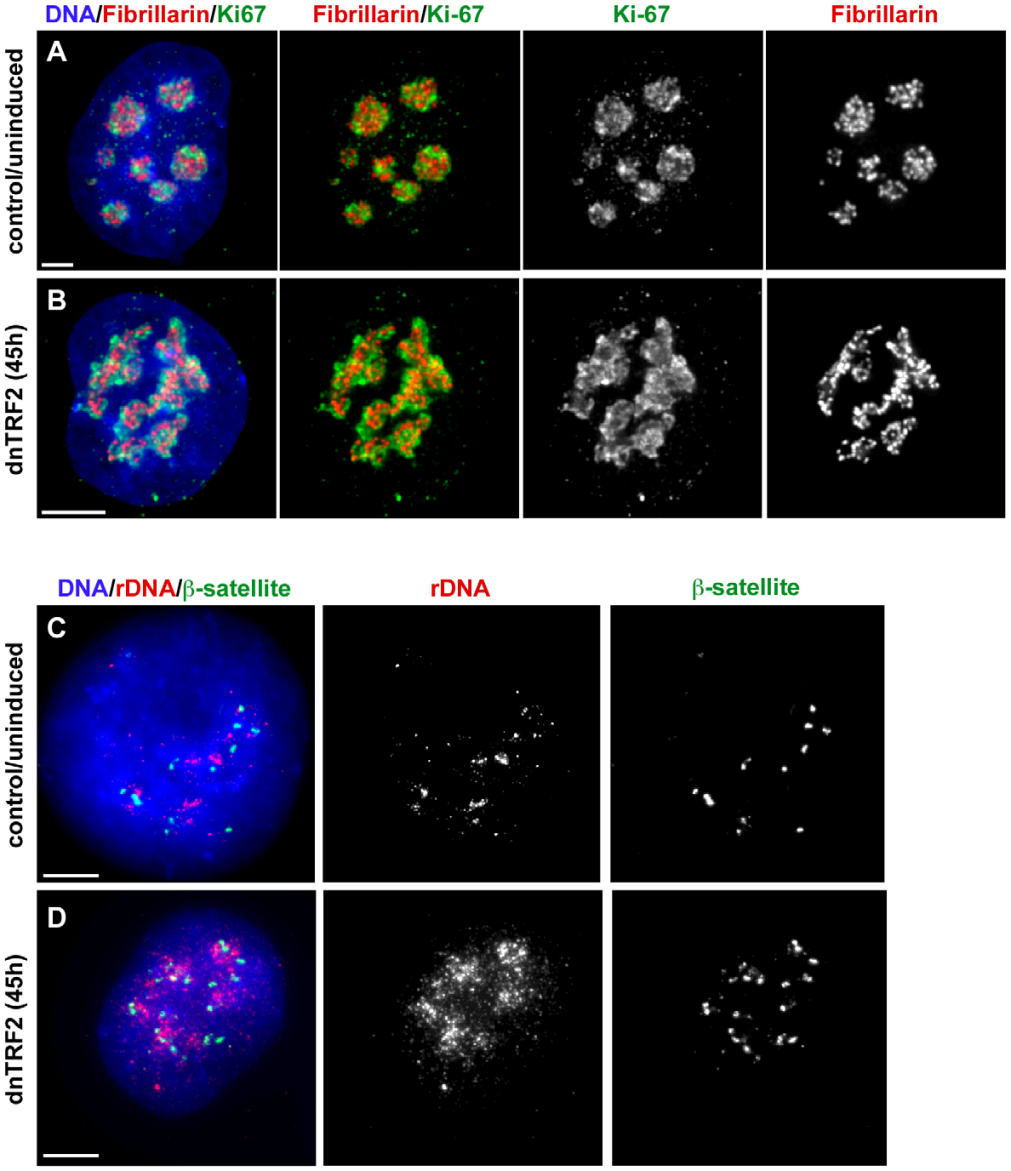
dnTRF2 expression alters nucleolar and acrocentric short arm architecture. Nucleoli in 3-D preserved cells isolated after 45 hours of dnTRF2 expression were identified in intact nuclei using immunostaining for fibrillarin (red) and Ki-67 (green). (A) In control (uninduced) cells, the nucleolus appeared as multiple punctate lobes in which fibrillarin and Ki-67 were intertwined. (B) In cells expressing dnTRF2, nucleolar morphology was abnormal, appearing as unraveled “nucleolar necklaces” rather than punctate structures in the center of the nucleus. (C) FISH with ß-satellite and rDNA probes on interphase nuclei showed that acrocentric repeat arrays normally appeared as tightly compacted foci. (D) ß-satellite (green) and rDNA (red) arrays were widely dispersed throughout the nucleus after expression of dnTRF2 for 45 hours, indicating that telomere dysfunction induced by mutant TRF2 disrupted normal nuclear organization. Scale bars for all panels = 5 µm.

To better understand mechanisms of iROB formation in our experimental assay, we considered circumstances under which the five pairs of acrocentric chromosomes preferentially interact. The tandem arrays of ribosomal RNA gene (rDNA) clusters or nucleolar organizing regions (NORs) are present on each acrocentric short arm. After mitosis, numerous mini-nucleoli arise around the NORs, coalescing into one or a few large nucleoli during interphase that bring the acrocentric short arms into close proximity [10], [55]–[58]. To test if dnTRF2 perturbed acrocentric associations, we visualized nucleolar organization in control (HT1080 and uninduced cells) and dnTRF2-expressing cells by immunostaining for two nucleolar proteins, Ki-67 and fibrillarin on three-dimensionally preserved nuclei (Figure 4A and 4B). HT1080 cells were also studied to exclude the possibility that the presence of the inducible mutant TRF2 construct, even when transcriptionally repressed, affected nucleolar and acrocentric organization. After 36–45 hours of dnTRF2 expression, Ki-67 immunostaining appeared ruffled rather than tightly compacted when compared to control nuclei (Figure 4A and 4B). Fibrillarin antibody staining was even more dramatically altered in dnTRF2 cells (Figure 4A and 4B). It was dispersed throughout the nucleus such that the nucleolus appeared unraveled. The morphology was reminiscent of “nucleolar necklaces” that have been previously described in cells treated with RNA polymerase II inhibitors [59], [60]. These experiments indicated that dnTRF2 profoundly affected nucleolar integrity.

Since nucleolar assembly depends on rDNA within the acrocentric short arm, we also examined the shape and positioning of rDNA and ß-satellite DNA arrays within interphase nuclei. In control nuclei, the arrays appeared as multiple punctate foci (90%, n = 30) (Figure 4C). After dnTRF2 was expressed for 45 hours, rDNA and ß-satellite FISH signals were diffuse and unraveled (Figure 4D), particularly rDNA. Over 85% of nuclei exhibited dispersed or scattered rDNA and ß-satellite signals. Collectively, these experiments suggest that dnTRF2 expression not only destabilizes telomeres, but leads to nucleolar disruption, short arm satellite instability, and DNA damage within the acrocentric short arms.

### Several mitotic and centromeric fates are associated with induced dicentrics

**Figure 5:**
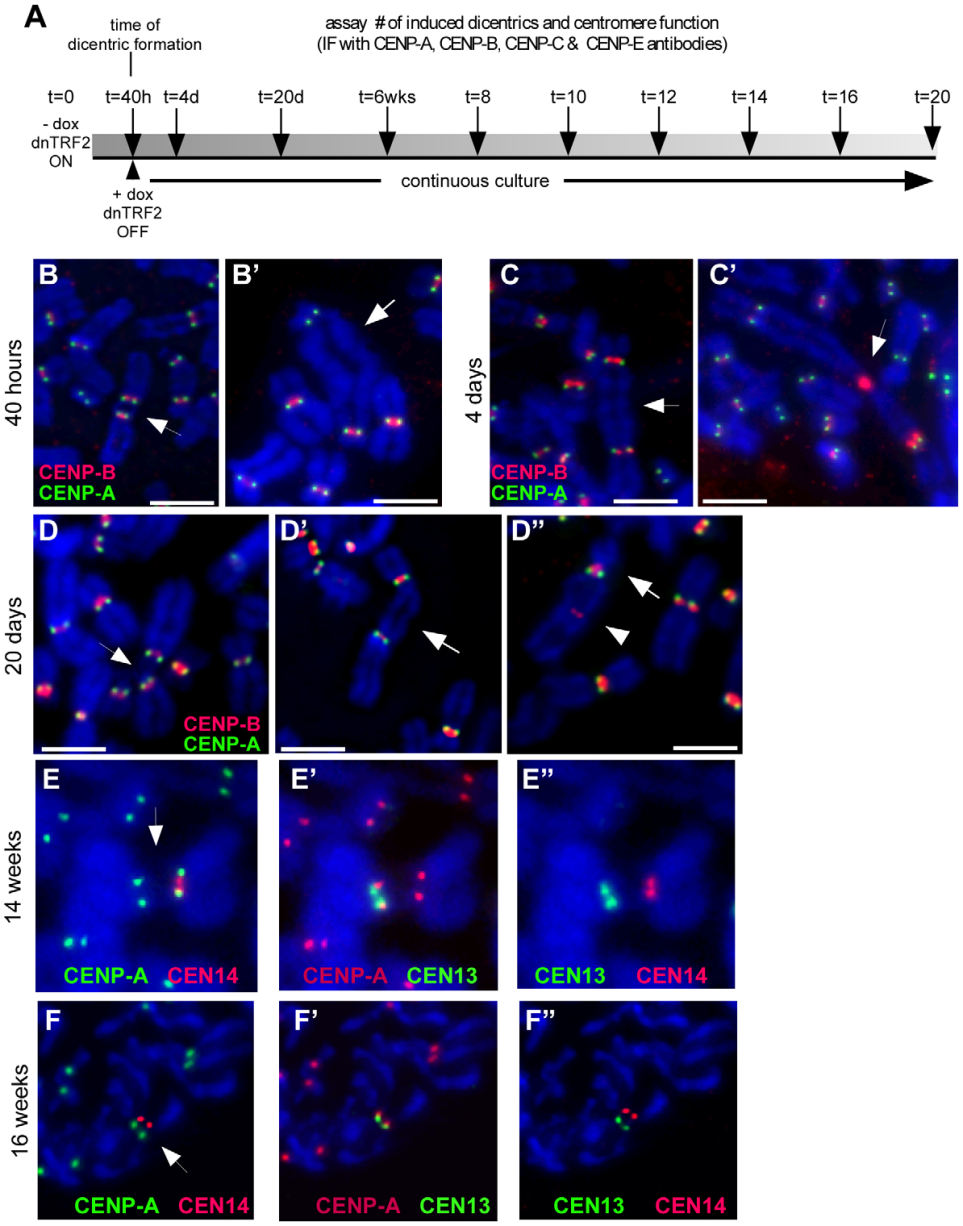
Centromere function of induced dicentrics. (A) Scheme of experimental strategy to produce *de novo* dicentrics for which centromere function and mitotic stability were monitored every 2 weeks (wks) for a total of 20 weeks. Centromere function was assayed by immunostaining for various centromere proteins (CENPs). Centromeric DNA regions were identified using CENP-B immunostaining or FISH with α-satellite–specific DNA probes. (B) Assessment of centromere function using immunostaining for CENP-A (green) and CENP-B (red). CENP-A identifies functional centromeres. CENP-B is an α-satellite DNA binding protein that binds to both active and inactive centromeres. After 40 hours (40h) of dnTRF2 expression, dicentrics were formed, including iROBs (B) and dicentrics involving non-acrocentric chromosomes (B′). Each type of dicentric, denoted by arrows, had two active centromeres. Scale bars = 7.5 µm. (C) At 4 days (4d) after dicentric formation, functionally dicentric chromosomes were still observed, including on chromosomes with two distantly located centromeres (C, arrow). Some cells also contained structurally tricentric chromosomes, as shown in (C′, arrow). In this case, one centromere was inactivated, since it exhibited immunostaining for CENP-B only. Scale bar in C = 5 µm; in C′ = 7.5 µm. (D) Several types of functionally dicentric chromosomes persisted even at 20 days (20d) after formation. These included iROBs (D) and non-acrocentric dicentrics with large inter-centromeric distances (D′). However, some functionally monocentric dicentrics were observed (D″), primarily among the non-acrocentric class of dicentrics. Arrows denote the chromosome fusion in each panel. Scale bars = 7.5 µm. (E) Assessment of centromere function on an irob(13;14) after 14 weeks of continuous culture (∼100 cell divisions). Centromeres were detected using FISH with α-satellite probes. CENP-A staining (green) appeared at the CEN14 (E, red). (E′) CENP-A (red) was also present at CEN13 (green), indicating that this iROB was functionally dicentric. (E″) This image shows the same iROB detected only with FISH probes to illustrate that CEN13 (green) and CEN14 (red) were spatially distinct. (F) Functionally monocentric iROBs were also detected during the timecourse experiment. Another irob(13;14), different than the one in (E), showed evidence of centromere inactivation. In (F), CENP-A (green) did not overlap with CEN14 (red). (F′) CENP-A (red) and CEN13 (green) co-localized, indicating the CEN13 was the functional centromere and CEN14 had been inactivated. (F″) This image shows the same iROB detected only with CEN13 (green) and CEN14 (red) FISH probes to illustrate that the centromeric arrays were spatially distinct.

**Figure 6:**
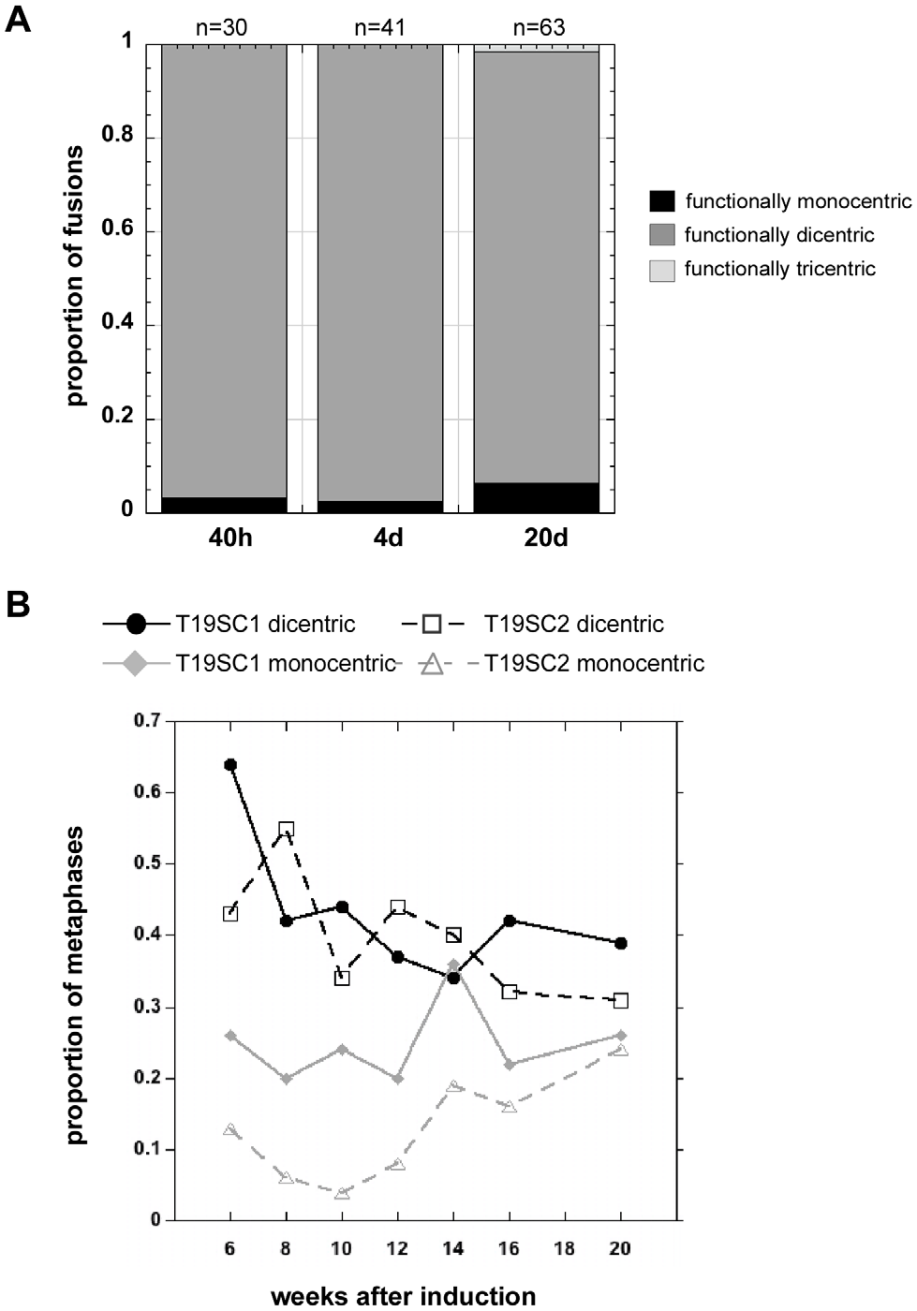
Induced dicentrics are variably stable over time. (A) The number of functionally monocentric, dicentric and tricentric chromosomes was monitored for 3 weeks after transient dnTRF2 expression. At 40 hours (40h) and after 4 days (4d) of continuous culture, nearly all structurally dicentric chromosomes remained functionally dicentric. By 20 days (20d), there was a noticeable increase in the proportion of functionally monocentric dicentrics. A functionally tricentric chromosome was seen at 20d. “n” represents the number of multicentrics (2+ centromeres) at each time point. (B) In longer-term experiments extending over a 14-week period, the proportion of functionally monocentric versus functionally dicentric iROBs was determined for subclones T19SC1 and T19SC2. These clonal lines were derived from two independent dnTRF2 short-term (36 hour) inductions. Overall, the number of functionally dicentric iROBs decreased (T19SC1) or stayed constant (T19SC2). The number of functionally monocentric iROBs increased in T19SC2. It should be noted that many cells contained more than one dicentric so that proportions will not add up to 1. Between 25 and 126 cells were scored at each timepoint for each cell line.

An advantage of the *in vitro* assay is the ability to monitor centromere function and dicentric stability immediately after formation. We used immunostaining for various centromere proteins (CENPs) to evaluate centromere function over time (Figure 5A) [26], [61]. It is important to note that in each induced line, every cell contained one or more different dicentrics. We analyzed this heterogeneous population of dicentrics that were formed after 40 hours of dnTRF2 expression. Cells were then returned to dox+ media and analyzed again after 4 days and 20 days of continuous culturing (Figure 5A). At 40 hours (i.e. time of dicentric formation), >95% of dicentrics, including iROBs with closely spaced centromeres (∼20Mb or less) and non-acrocentric dicentrics in which centromeres were distantly located (estimated to be >50Mb apart), had two functional centromeres (Figure 5B, Figure 6A). Only one dicentric showed CENP-A at one of the two α-satellite regions, suggesting that centromere inactivation had occurred soon after formation (Figure 6A). Even after 4 days of continuous culture (∼4 cell divisions), the number of functionally dicentric chromosomes was unchanged (97%) (Figure 6A). These dicentric chromosomes included iROBs and non-acrocentric dicentrics. In fact, 80% of non-acrocentric dicentrics were functionally dicentric, including those with large inter-centromeric distances (Figure 5C and 5C′). Centromere inactivation was observed at 4 days, but in a minority of fusions (Figure 6A). For instance, a structurally tricentric chromosome lacked CENP-A at one of its three centromeres (Figure 5C′). The centromeres that remained active were located at opposite ends of the chromosome.

At 20 days after dicentric formation, the number of functionally monocentric chromosomes increased from 2–3% to 6% (Figure 5D″), and the number of functionally dicentric chromosomes decreased slightly (Figure 6A). Nevertheless, even after ∼20 cell divisions, both functionally dicentric iROBs and non-acrocentric fusions were still observed (Figure 5D and 5D′). A functionally tricentric chromosome was even observed (Figure 6A). We conclude that human chromosomes with two (or more) active centromeres are mitotically stable for many cell divisions after their formation. In patient-derived dicentrics, centromere distance is thought to influence whether a dicentric has one or two active centromeres [16], [62]. Our experimental data support a model for centromere function on newly-formed dicentric chromosomes that is less dependent on centromere distance. Clearly, *de novo* dicentric behavior is more complex than previously appreciated from studies of patient-derived dicentrics.

To monitor longer-term dicentric behavior, the time course experiments were extended. First, the population of dicentrics was enriched for the most prevalent dicentrics. This was achieved by sub-cloning the induced cell lines for several weeks to yield cell lines that contained only a few types of iROBs. At 6 weeks after formation, many induced iROBs had undergone centromere inactivation, but at least half remained functionally dicentric (Figure 6B). We then monitored the number of functionally monocentric and dicentric iROBs in two subclones, T19SC1 and T19SC2, over the next 14 weeks. We observed functionally dicentric iROBs even after 14 weeks of continuous division (∼100 cell divisions) (Figure 5E–5E″, Figure 6B), although some iROBs underwent centromere inactivation (Figure 5F–5F″, Figure 6B). During the 14 weeks, the proportion of functional dicentrics in clone T19SC1 decreased, and in clone T19SC2, the number of monocentrics increased from 15% to nearly 25% (Figure 6B). Since we evaluated cells that contained more than one iROB and only a subset of cells from the population were assayed at each timepoint, it was important to consider the trend across the entire timecourse. In general, the proportion of functionally dicentric chromosomes did not increase in either clone, and functionally monocentric chromosomes either remained consistent or increased. These results indicated that centromere inactivation occurs within several weeks after dicentric formation. However, many newly formed dicentrics remained functionally dicentric for ∼180 cell divisions.

### *De novo* dicentrics exhibit variability in functional centromeric state

A caveat of the previous experiment is that it broadly evaluated all dicentrics in the cell populations of T19SC1 and T19SC2. Thus, we monitored centromere function of specific iROBs. These included two independent versions of irob(13;14), an irob(13;13), and an irob(14;14). Other iROBs, such as an irob(15;22) and several irob(22;22), were also evaluated (data not shown). Centromere function was analyzed every 2 weeks by immunostaining for CENP-A, CENP-C, and/or CENP-E (Figure S5A). We observed iROBs that remained functionally dicentric (Figure 5E–5E″), while others underwent inactivation (Figure 5F–5F″). Even iROBs involving the same acrocentrics behaved differently over the 14-week period. At the starting point of the timecourse (6 weeks after formation), one irob(13;14) was functionally monocentric (Figure S5A) and the other was functionally dicentric (Figure S5B). However, both were functionally dicentric in most or all cells at 20 weeks (Figure S5A and S5B). Conversely, other iROBs showed evidence of centromere inactivation. For instance, an irob(13;13) had already undergone inactivation in half the cells at 6 weeks, but was functionally monocentric in most cells by 14 weeks (Figure S5C). The irob(14;14) was functionally dicentric at 6 weeks, but had undergone inactivation in nearly all cells at 20 weeks (Figure S5D). These experiments revealed notable differences in centromere function of iROBs, although the molecular basis is still unclear. It is unlikely that the identity of the chromosomes involved in the iROB determines its fate since this experiment revealed that the same type of iROB could behave differently over time. For instance, one irob(13;14) (Figure S5B) was present as a functional dicentric over the entire time course, but the other irob(13;14) existed in both functionally dicentric and functionally monocentric states within the same cell population (Figure S5A). This might reflect instability of centromere function on the same iROB over time, hierarchical centromere disassembly, or differences in timing of inactivation in each cell. This set of experiments provides evidence that *de novo* structurally dicentric human chromosomes are mitotically stable in either functionally monocentric or functionally dicentric configurations. The timeframe during which centromere inactivation happens is broad, occurring several weeks to months after dicentric formation.

### Centromere inactivation by deletion of centromeric (α-satellite) DNA

**Figure 7:**
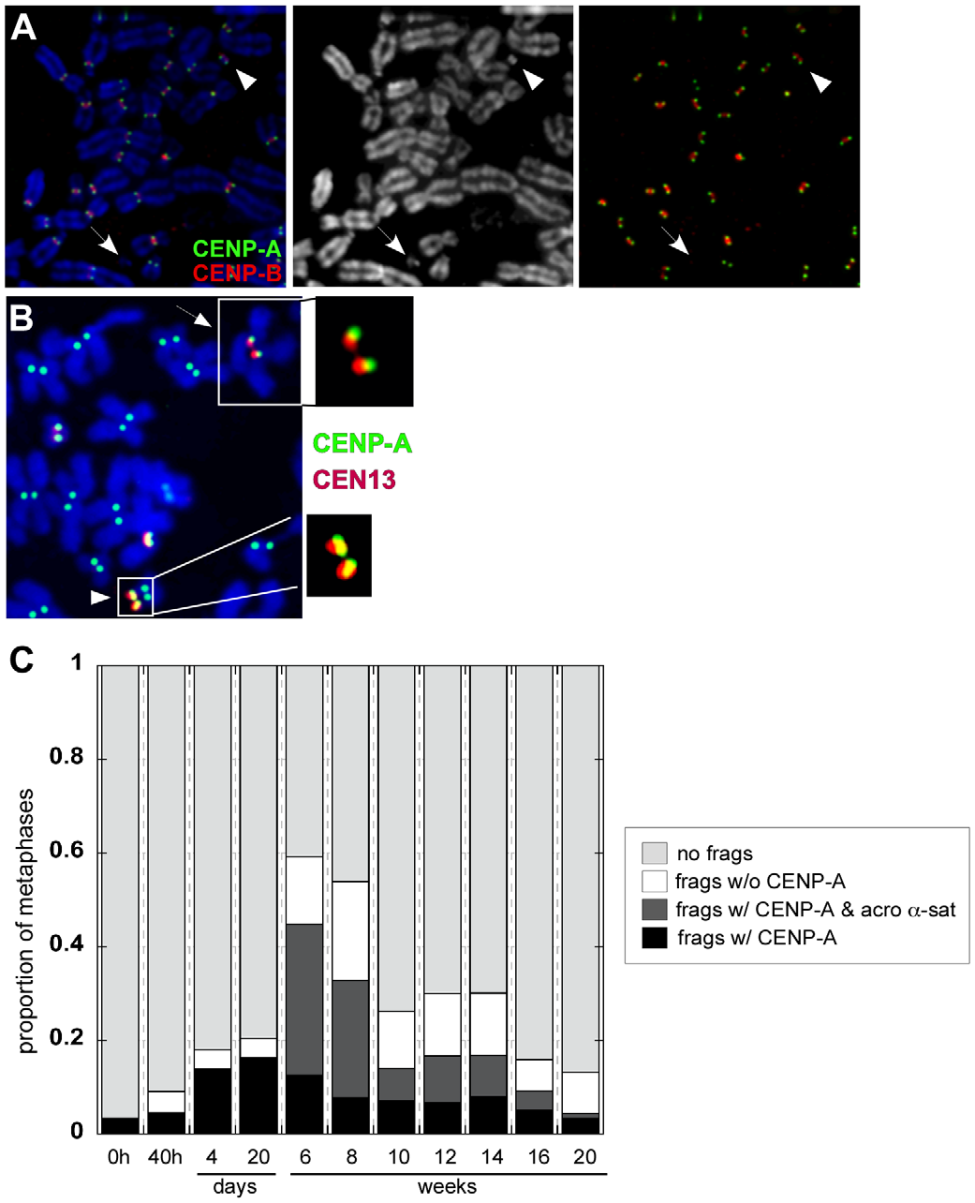
Dicentric stability is associated with chromosomal fragments. (A) The appearance of small chromosome fragments either containing or lacking CENP-A (green) and CENP-B (red) was monitored over time, from the time of dicentric formation (40h) to 20 weeks. Arrowheads denote chromosome fragments. The DAPI image is shown in the middle panel. Combined CENP-A (green) and CENP-B (red) is shown in the far right panel. (B) At 16 weeks after formation of an irob(13;14), immunostaining for CENP-A followed by FISH with chromosome-specific α-satellite probes revealed that CENP-A did not co-localize with CEN13 (arrow; see enlargement of centromeric region). A small CEN13 fragment that was CENP-A-positive (arrowhead; see enlargement of CENP-A/α-satellite signal of fragment) was present in the same cell. This chromosomal fragment was hypothesized to have originated from the inactivated CEN13 of the iROB. (C) Chromosomal fragments, with and without CENP-A and acrocentric α-satellite DNA was monitored over time. CENP-A-positive chromosome fragments (black+dark gray bars), many of which corresponded to acrocentric α-satellite DNA (dark gray), were prevalent after dicentric formation. These fragments decreased after 20 weeks, suggesting that they were lost during cell division. Thirty to 270 cells per time point were analyzed.

Current models for centromere inactivation, derived from studies in yeast and maize, implicate centromeric deletion or chromatin remodeling at one centromere of a dicentric [63], [64]. Experimental evidence for the molecular mechanism(s) of centromere inactivation in humans has been limited [65], [66]. Between 4 days and 20 weeks after dicentric formation, we detected small chromosomal fragments in many of the same cells that contained iROBs. Many fragments showed CENP-A antibody staining (Figure 7A). To investigate their genomic origin, we used combined CENP-A immunostaining and FISH to evaluate cells between weeks 6 and 20. Approximately 60% of all the CENP-A positive fragments contained acrocentric α-satellite sequences (Figure 7B and 7C). The non-acrocentric fragments were most likely derived from other α-satellite arrays or non-centromeric regions that were lost when large dicentrics underwent breakage. The number of fragments increased by 4 days after dicentric formation, peaking at 6 weeks, and then decreased thereafter (Figure 7C). As the number of functionally dicentric chromosomes decreased over time, the proportion of chromosome fragments also declined. In many cells that contained both CENP-A/α-satellite fragments and a dicentric chromosome, the dicentric had undergone inactivation and the identity of the chromosomal fragment corresponded to the inactivated centromere (Figure 7B). Our interpretation of these results is that during centromere inactivation, partial deletion of one α-satellite array occurred. Since α-satellite DNA but not CENP-A was detected at the inactivated centromere, we propose that deletion removed the region of chromatin containing CENP-A nucleosomes.

CENP-A is assembled on only a portion (30–50%; 0.2–2Mb) of the multi-megabase arrays of α-satellite [38], [40] (L.L. Sullivan et al., unpublished data). Removal of the CENP-A portion of an α-satellite array should measurably reduce total array size. To test this hypothesis, we used a quantitative FISH approach in which the intensity and number of pixels from a fluorescent probe hybridized to a centromere is correlated to the size of an α-satellite array (Figure S6) [67], [68]. Specifically, we measured centromeric probe intensities for two independent irob(14;21)s. Molecular studies of α-satellite array sizes were considered difficult and most likely inconclusive, since multiple acrocentrics share the same sequence (i.e 13/21 and 14/22), and there were 3–4 copies of each acrocentric chromosome in the HTC lines. An advantage of the cytological approach is that the chromosomes could be visually identified so that the same centromere and chromosome could be studied before and after dicentric formation. Furthermore, the HSA21 homologues of the HTC lines were easily distinguishable. One pair exhibited a bright, large α-satellite FISH signal (CEN21^L^), while the other pair had a small FISH signal (CEN21^S^) (Figure S6). There were also 4 copies of HSA14 in the cells, but the FISH signals appeared the same, suggesting that the α-satellite arrays were similarly sized. However, one HSA14 was structurally abnormal, and contained duplicated material on the distal q arm. We could easily exclude this HSA14 from our analyses, since it was not involved in either irob(14;21).

**Figure 8:**
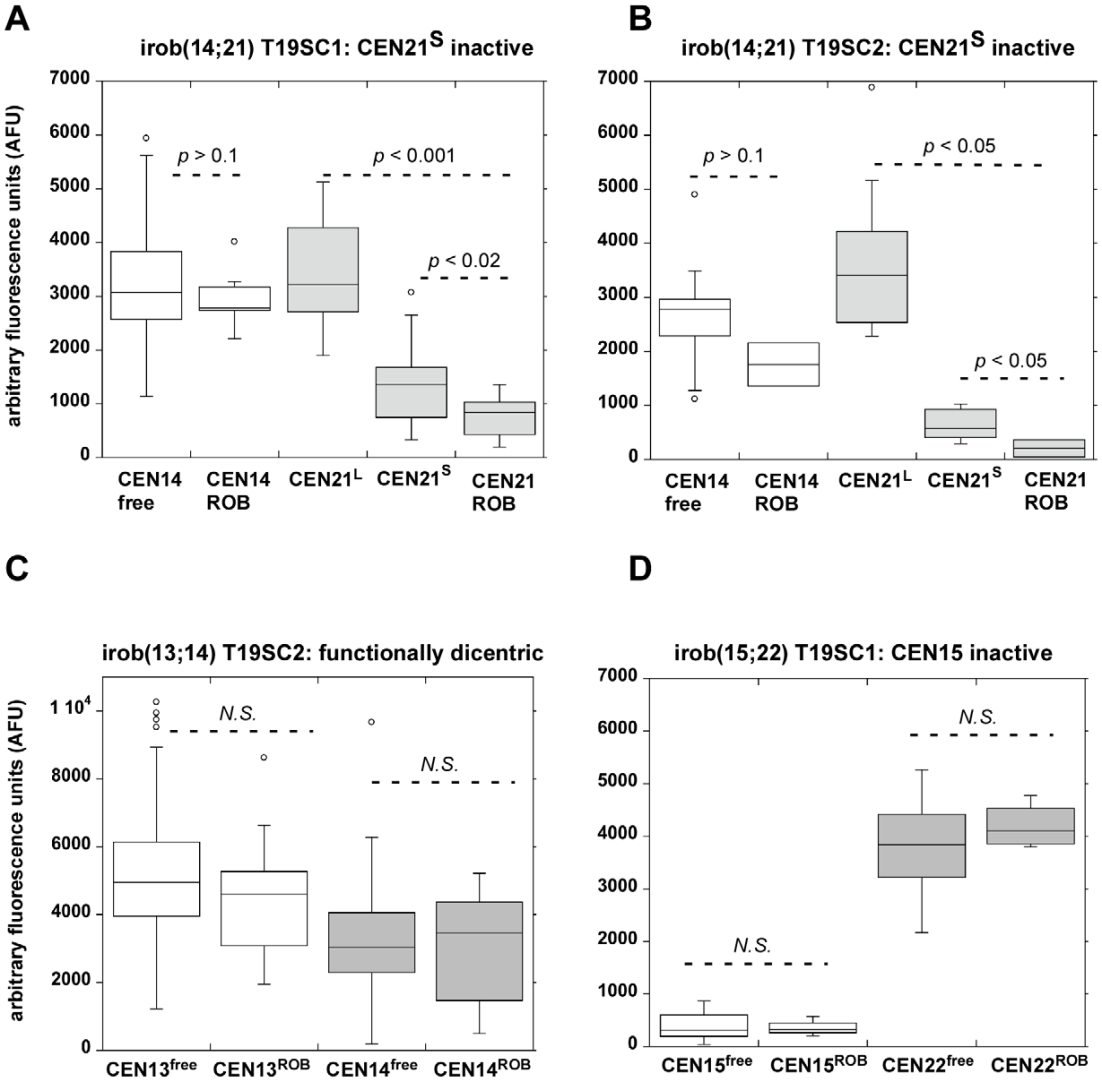
Reduction in α-satellite DNA FISH signals suggests that partial centromeric deletion occurs at inactivated centromeres of iROBs. Metaphases from control cells and from induced subclones T19SC1 and T19SC2 containing different versions of specific iROBs [irob(14;21) and irob(13;14)] were hybridized with chromosome-specific centromeric (α-satellite) probes. The integrated densities of the fluorescent α-satellite signals were measured in multiple cells on free-lying acrocentrics and the same chromosome after iROB formation. Fluorescence intensities in arbitrary fluorescence units (AFU) were displayed as box plots. The centromeres of the HSA21 homologues were visually distinct, in that one pair of homologues had a large α-satellite FISH signal, designated as CEN21^L^, while the other pair of homologues had a much smaller FISH signal (denoted as CEN21^S^) (see Figure S6 for additional information on identification of HSA21 homologues). *P* values indicating significant differences in fluorescence intensities were determined using the Mann-Whitney test. (A, B) In two independent versions of irob(14;21), CEN21 was identified as inactive by CENP-A immunostaining. Specifically, one CEN21^S^ homologue appeared to be involved in the irob(14;21). The range of AFUs at the inactive CEN21 on the iROB was decreased compared to either free-lying CEN21^L^ or CEN21^S^, suggesting that CEN21^ROB^ had become smaller during or after iROB formation. The intensity of the CEN14 probe at the active CEN14 of the iROB (CEN14^ROB^) was not significantly (N.S.) different from CEN14 on free-lying HSA14 (CEN14^free^). (C) As validation for the quantitative assay, a functionally dicentric irob(13;14) was examined. Fluorescence intensities of centromeres on free-lying HSA13 (CEN13^free^) and on the iROB (CEN13^ROB^) were not statistically different. Similar fluorescence intensities were also observed when CEN14 FISH was compared on free-lying HSA14 and the irob(13;14). *N.S.* = not significant, *p*>0.1. (D) A different type of iROB, irob(15;22) in which CEN15 was inactive was analyzed. In this iROB, a significant difference between CEN15^free^ and CEN15^ROB^ was not detected, suggesting that inactivation had occurred by an epigenetic mechanism or deletion was below the level of FISH detection. *N.S.* = not significant, *p*>0.1.

The fluorescence intensities of FISH probes for the centromeres of the free-lying acrocentric chromosomes were measured in control (uninduced) lines (Figure S6). Then the CEN14 and CEN21 signals were measured on the irob(14;21), and compared to signal intensities from the free-lying centromeres in control cells. Since the CEN21 signals from the two sets of HSA21 homologues were distinctive, it was obvious which HSA21 was involved in both iROBs. In both irob(14;21)s, the HSA21s with CEN21^L^ remained free-lying (Figure S6), implying that one HSA21 with CEN21^S^ was involved in the iROB. CEN21^ROB^ FISH signal intensities were significantly smaller than both CEN21^L^ and CEN21^S^ (*p*<0.05), while active CEN14 intensities were unchanged (*p*>0.1) (Figure 8A and 8B). As controls for this assay, we measured α-satellite intensities on two independent irob(13;14)s that were functionally dicentric. The fluorescence intensities of CEN13 and CEN14 before and after dicentric formation were similar (Figure 8C and data not shown). Partial α-satellite deletion may not be the only mechanism of inactivation since one irob(15;22) did not exhibit any differences in α-satellite FISH intensities/array size between CEN15 before and after its involvement in the iROB (Figure 8D). This centromere was very small on the free-lying HSA15, so perhaps there is a size threshold below which centromeric deletion is less likely to occur. We conclude that CEN15 is inactivated via an epigenetic-dependent mechanism, which is in agreement with studies of other human dicentric chromosomes [69]. Overall, these experiments suggest several mechanisms of centromere inactivation, one of which involves deletion of α-satellite DNA associated with CENP-A.

## Discussion

Dicentric chromosomes, while unstable in many organisms, are quite stable in human cells, although a selection bias for the most stable dicentrics may contribute to this perception. Here we report a system in which telomere de-protection induced by transient removal of TRF2 non-randomly produces *de novo* chromosome fusions *in vitro*, resulting in a high proportion of acrocentric fusions. Our investigation thus revealed parallels between the identity of prevalent naturally-occurring human translocations and experimentally-produced rearrangements. The prevalence of induced ROBs (iROBs) suggests an inherent property of acrocentric chromosomes that predisposes them to rearrangement and fusion. Our data argue that spatial relationships within the interphase nucleus promote the non-random fusions observed in our assay. Acrocentric chromosomes are located near each other during the cell cycle when the nucleolus forms around the nucleolar organizing regions (NORs), or arrays of ribosomal RNA genes, located on each acrocentric short arm. In the inducible dnTRF2 system, chromosome ends that have lost endogenous TRF2 and are closely located would be most likely to fuse versus chromosomes located far apart. Since the acrocentrics cluster at the nucleolus, they may have a greater chance of fusing to each other. However, any given chromosome is located next to others, so the number of non-acrocentric fusions might be predicted to offset the number of acrocentric fusions. Nevertheless, non-acrocentric fusions were significantly under-represented. Thus, proximity of the acrocentrics and the bias for these fusions when dnTRF2 is expressed may be coupled to nucleolar dynamics and/or DNA damage and repair within acrocentric short arms.

### Telomere dysfunction affects stability of neighboring sequences

After dnTRF2 induction, unprotected acrocentric chromosome ends would appear as double-strand breaks (DSB)s to be repaired by non-homologous end joining (NHEJ), presumably using the nearest neighbor which would be another acrocentric. Surprisingly, one-fifth of the iROBs lacked visible telomeric repeats at their fusion points, suggesting an alternative or more complex mechanism of fusion. Although small amounts of telomeric repeats might be present below the level of FISH detection, the absence of repetitive sequences immediately adjacent to the telomere provided compelling evidence that telomeric DNA, as well as other acrocentric short arm DNA, had been deleted during dicentric formation. How the DNA damage is repaired is not clear. Heterogeneity in the amount of short arm repeats retained on each iROB suggests a mechanism of NHEJ. However, we cannot discount that more complex mechanisms of homologous or heterologous recombination are also involved. Studies in other organisms have illustrated that NHEJ, recombination, or break-induced replication (BIR) can result in compound genomic signatures on end-to-end fusions and dicentric chromosomes [20], [70]–[72]. In our system, once dicentrics formed, acrocentric short arm composition did not noticeably change, even after months in culture, arguing against a model of molecular heterogeneity due to ongoing breakage, re-fusion and reorganization of iROB short arms. Although small rearrangements might have existed below the detection of FISH, the most consistent interpretation of our data is that in addition to telomere function, mutant TRF2 impacts acrocentric short arm stability. A major challenge of this experimental system is that inducible, parental cell lines contain >15 acrocentric chromosomes, and molecular identification of precise breakpoints is difficult due to shared satellite DNA homologies and complicated arrangements of repeat blocks.

### Nucleolar disruption by dnTRF2: cause or effect of acrocentric instability?

Abnormal nucleolar morphology and dispersal of acrocentric short arm satellite repeats in nuclei of dnTRF2-expressing cells suggested a potential extra-telomeric role for TRF2. TRF2 is transiently associated with the nucleolus during the cell cycle, and when its release is prevented by the RNA polymerase inhibitor actinomycin D, chromosome end-to-end fusions occur [73]. Movement of TRF2 to and from the nucleolus may ensure proper nuclear and chromosome architecture, although it is unclear if TRF2 directly interacts with acrocentric DNA and/or nucleolar proteins or regulates rDNA transcription. Acrocentric telomere sequences cluster around the nucleolar periphery [74], so simple proximity of the telomere to the acrocentric short arms may explain why TRF2 is detected at the nucleolus. Still, TRF2 has been reported to bind at non-telomeric sites on acrocentric short arms, near sites of upstream binding factor (UBF) and B23/nucleophosmin [73]. UBF and other nucleolar proteins from the previous cell cycle form the nucleolus in the subsequent cell cycle [75], so dnTRF2 may disrupt putative TRF2-UBF interactions, UBF-acrocentric arm associations, or even TRF2-acrocentric DNA interactions. It remains to be determined if nucleolar disruption results from dnTRF2-induced telomere dysfunction, unstable acrocentric short arm sequences, or formation of multiple acrocentric fusions that lack rDNA. We have observed that acrocentric fusions form non-randomly in human cells transiently expressing exogenous Cre recombinase (K.M. Stimpson and B.A. Sullivan, unpublished observation). In these cells, nucleolar organization and assembly remain intact, so we conclude that iROB formation can be discounted as the primary mechanism responsible for nucleolar defects observed in the present study.

### Dicentric stability achieved after a period of instability

Dicentric chromosomes in many organisms undergo classical breakage-fusion-bridge cycles [19], [21]. For instance, engineered *Drosophila* dicentrics are unstable and break during mitosis [20], [76], although they can segregate accurately in female meiosis [77]. In budding yeast, dicentric plasmids or linear chromosomes are also unstable, but become less so under conditions in which one centromere is deleted, inter-centromeric distances are decreased, or transcription is forced through the centromere [43], [63], [78]. Centromere inactivation has not been described to occur naturally in yeast, but is more frequent in human dicentrics [62], [66]. As such, former conclusions drawn about dicentric instability in model organisms may not have revealed similarities in dicentric behavior between plants and mammals. Indeed, recent studies in plants have suggested that centromere inactivation occurs at formerly under-appreciated frequencies [64]. In these studies, dicentric stability was accompanied by chromosome breakage, a phenomenon that we also observed in our study. We also observed centromeric deletion as a mechanism of dicentric stabilization. Our studies emphasize several parallels in dicentric behavior among model organisms and humans.

### Functional dicentrics and the influence of inter-centromeric distance

An aspect of dicentric behavior that appears to be unique to humans is the observation that dicentrics often exist as functionally dicentric chromosomes [62], [66], [79]. In patient-derived dicentrics (i.e. dicentric Xs and many *de novo* ROBs) short inter-centromeric distances have been proposed to influence centromere function, so that dicentrics with closely spaced centromeres are more likely to remain functionally dicentric. However, over 80% of patient-derived ROBs undergo centromere inactivation and even dicentric Xs with closely spaced centromeres experience centromere inactivation [16], [26], [80]. So do inter-centromeric distance and dicentric centromere function correlate exactly or randomly? Our present study explored this question, as the largest estimated distance between the centromeres of some iROBs was ∼20Mb. Such short inter-centromeric distances might explain why functionally dicentric iROBs were maintained for up to 6 months. Nevertheless, about half of the iROBs underwent centromere inactivation, even those in which centromeres were maximally separated. Even more compellingly, fusions involving non-acrocentric chromosomes also remained functionally dicentric (or tricentric) for up to 20 cell divisions. The centromeres were estimated to be at least 50Mb apart, yet these dicentrics were retained for several weeks. In fact, extensive chromosome fragmentation or breakage was not observed until 6 weeks after dicentric formation. Our studies of *de novo* dicentrics argue that centromeric distance is not the strongest predictor of the functional state of a dicentric chromosome. Centromere inactivation in iROBs might rely instead on chromosome-specific features or may occur differently in each cell. It is possible that induced dicentrics with larger inter-centromere distances eventually undergo inactivation, as observed in patient-derived dicentrics, or experience breakage as predicted from model organisms. Future long-term studies using this and other experimental systems should address these questions in more detail.

### The temporal nature of centromere inactivation

We observed that centromere inactivation occurred 2–20 weeks after dicentric formation. This timeframe is consistent with studies of maize dicentric chromosomes that undergo centromere inactivation at 10 weeks after formation [22], [64]. However, dicentrics can also exhibit more complex patterns of centromere function. Dicentric human chromosomes are sometimes present in both functionally dicentric or monocentric states within the same individual or cell line [62], [66]. Some induced dicentrics also exhibited this behavior, and in these cases, the same centromere lacked CENP-A, -C or -E staining when the dicentric was in the functionally monocentric configuration. Centromeres have been reported to change functional states in both fission yeast and clonal lines of isodicentric Xs [66], [69], [81]. Centromere switching in human dicentrics has been defined as the presence of the dicentric in functionally monocentric and dicentric states and is considered more prevalent in dicentrics that have genomically identical centromeres [69]. However, we observed variable centromere states in different cells and at different times for induced dicentrics with non-homologous centromeres. The biological mechanism for this phenomenon is unclear. Perhaps inactivation had not occurred completely at a given assay point. Centromere disassembly may occur in stages and centromere proteins for which we had not assayed might have been maintained on some versions of the iROBs. A model for hierarchical centromere inactivation in which centromere proteins are lost sequentially has emerged from a recent study of conditional centromeres on human artificial chromosomes [82]. Alternatively, timing of inactivation might vary among cells, so that our observations reflect differences in the number of cells containing a dicentric that had or had not undergone inactivation. Future experiments are required to distinguish between models of centromere switching, incomplete centromere inactivation, or cell-specific differences in the timing of centromere inactivation.

### Deletion as a mechanism of centromere inactivation in dicentric human chromosomes

Genomic deletion has been implicated in inactivation of yeast dicentrics and inferred from studies of patient-derived dicentric Y chromosomes [65], [67]. However, since this is not the case for dicentric X chromosomes [69], it was unclear if such a mechanism might be yeast or Y-specific. Our study provides the first experimental evidence that newly-formed dicentrics in human cells can be stabilized by centromeric deletion. A notable difference between this type of mechanism in yeast and humans is that α-satellite DNA, the genomic marker of the human centromere, was not completely removed during inactivation. Instead, only the portion associated with CENP-A and what presumably identifies the site of kinetochore assembly, appeared to be eliminated (Figure S7). Spatial and temporal incorporation of the centromeric H3 variant CENP-A maintains the location and function of the centromere. CENP-A is at the top of the centromere assembly hierarchy, recruiting other centromere and kinetochore proteins. Newly synthesized CENP-A is loaded into chromatin in late telophase/early G1 by the escort protein HJURP [83], [84]. Thus, removal of CENP-A, and other centromere/kinetochore proteins, from a centromere destined for inactivation must occur in addition to blocking the loading of new CENP-A. It is not known if existing CENP-A nucleosomes, recruitment of additional factors, or H3-containing nucleosomes within centromeric chromatin guide incorporation of new CENP-A or if such factors are recognized by HJURP or intermediates. It would be consistent with any of these models if centromere inactivation occurred by simultaneously deleting existing CENP-A and nearby accessory chromatin that target new CENP-A deposition.

This study provides evidence for a genomic mechanism of centromere inactivation that occurs in some dicentrics. Future studies will be important for determining the molecular basis for which inactivation pathway (genomic versus epigenetic) is taken. Events that initiate centromere inactivation and influence the fate of a dicentric may be triggered randomly, or by chromosome-specific features, such as α-satellite array size. The ability to produce *de novo* dicentrics in human cells should reveal additional insights into molecular mechanisms of centromere inactivation and dicentric behavior and offer a means by which centromere inactivation can be directly manipulated or perturbed.

## Materials and Methods

### Cell culture

HTC75 T19 and T4 clonal cell lines containing the Tet-inducible truncation allele of TRF2 (ΔBΔM) [44] were cultured in MEM alpha (Invitrogen) supplemented with 10% FBS, antibiotics (Invitrogen), 5 mM filter-sterile glucose, and 100ng/ml doxycycline hyclate (Fluka).

### Fixed metaphase isolation

Inductions of dominant-negative TRF2^ΔBΔM^ expression were carried out in doxycycline-free media for 24–40 hours, 3 days, and 5 days. Metaphase chromosomes were harvested using methanol∶acetic acid fixation (3∶1 v/v).

### Immunofluorescence

Metaphase spreads were prepared as previously described [26]. For IF on nuclei, cells were grown on glass slides. Cells were fixed in 4% paraformaldehyde in PBS and in the case of nuclei preps, permeabilized with PBS+Triton X-100. Antibodies included: mouse monoclonal anti-CENP-A antibodies (Abcam ab13939; 1∶500), rabbit polyclonal anti-CENP-A antibodies (Upstate 30217; 1∶200), rabbit polyclonal anti-CENP-B (Abcam 25734; 1∶400), mouse monoclonal anti-CENP-C (Abcam ab50974; 1∶200), mouse monoclonal anti-CENP-E antibodies (Abcam ab5093; 1∶200), mouse monoclonal to TRF2 (Imgenex; IMG-124A, 1∶200), mouse monoclonal to fibrillarin (Abcam ab18380; 1∶1000), rabbit polyclonal Ki67 (Novocastra; 1∶500), and rabbit polyclonal to gamma H2A.X phospho S139 (Abcam ab2894, 1∶400). Antibodies were detected with donkey anti-mouse or anti-rabbit secondary antibodies conjugated to Alexa Fluor 488 (Molecular Probes), Cy3 or Cy5 (Jackson Immunoresearch, Inc.).

### Probe preparation

ß-satellite repeats were detected using plasmid pß4 [8], satellite III DNA with plasmids pTRS-47 and pTRS-63, and HSA15 α-satellite DNA with pTRA-20. Biotinylated HSA15 satellite III probe (D15Z1) was from Oncor, Inc. Probes for 18s rDNA, HSA13/21 α-satellite, and HSA 14/22 α-satellite were created from cloned PCR products [85]–[87]. Plasmids were labeled with biotin-16-dUTP and digoxygenin-11-dUTP (Roche) by nick-translation. Whole-arm chromosome-specific DNA was amplified by PCR and labeled with biotin or digoxygenin [88] (Text S1). Telomere repeats were detected with biotin-labeled LNA probe (T_2_AG_3_)_3_ (Exiqon) or FITC-conjugated PNA probe (C_3_TA_2_)_3_ (Biosynthesis).

### Fluorescence in situ hybridization

FISH and IF-FISH were performed as described [26]. Metaphase chromosomes were denatured in 70% formamide/2× SSC pH 7 at 73°C for 2 minutes (conventional FISH) or at 80°C for 8 minutes (IF-FISH). Chromosome painting probe hybridization mixtures contained 10mg/mL Cot-1 DNA. Denatured Cot-1 DNA and painting probes were pre-annealed at 37°C after denaturation. Probes were detected with Cy3-, Cy5-conjugated anti-digoxin (Jackson Immunoresearch), or Alexa Fluor 488-streptavidin antibodies (Molecular Probes).

### Microscopy and image analysis

Images were acquired on an inverted Olympus IX-71 attached to the Deltavision RT restoration imaging system (Applied Precision, Inc.) equipped with a Photometrics CoolSNAP HQ CCD camera. Images were captured using the SoftWoRx Acquire 3D software using 40× (N.A. 1.35), 60× (N.A. 1.42), or 100× (N.A. 1.40) oil objectives (Olympus). Images were collected as *z*-stacks of 0.1–0.5mm increments (1–15 sections total), depending on the fixation technique. Image stacks were deconvolved using 10 iterations using a conservative algorithm, then collapsed using the Quick Projection option. Projections were converted to Adobe Photoshop for viewing and analysis.

### Quantitative FISH to assess centromere (α-satellite) array size reduction

Multi-color FISH was used to hybridize α-satellite probes recognizing the centromeres of HSA13/21, HSA14/22 and HSA15 to metaphase chromosomes from control cells (HT1080, HTC75 T19 uninduced) and 36-hour induction subclones T19SC1 and T19SC2. Digital images were collected using an epifluorescence microscope ensuring that no signal reached pixel saturation. Pseudo-colored three-color (RGB) images were separated into individual wavelengths for the green (488nm) and red (568nm) channels. Individual centromere signals from the same image were segmented by interactive intensity thresholding (via segmentation command) in IPLab/iVision (BioVision Technologies). The fluorescence intensity/integrated density (i.e. the sum of the values for all pixels within the region defined as the α-satellite array for each centromere) that corresponded to FISH signal intensity was measured in each segmented area/centromere. Integrated densities (ID) of centromeres on free-lying acrocentrics (in control and dnTRF2 cells) and iROB centromeres were collected from multiple images from the same experiment and compiled as arbitrary fluorescence units (AFUs). AFUs for the inactivated centromere were then compared to AFUs of α-satellite signal intensities for that particular centromere when on free-lying chromosomes.

### Statistical methods

Chromosomal interactions after dnTRF2 induction were plotted and displayed using Circos program [89]. Statistical significance of the proportion of fusions formed between induction timepoints was determined using a Students *t*-test at a confidence interval of 95%. Datasets including fluorescent signal intensities of centromeres on free-lying chromosomes and on chromosomes after iROB formation were tested for significance using parametric (t-test) and nonparametric tests (Mann-Whitney U test). Reported *P* values in Figure 8 were derived from Mann-Whitney tests. *P* values indicating the significance of specific chromosomal fusions were calculated using 2×2 contingency tables of observed versus expected ratios and the Chi-Square (χ^2^) test. The *t*-tests, Mann-Whitney U tests, and χ^2^ tests were performed using either Excel (Microsoft Corporation), Graph Pad Statistics software available online (http://www.graphpad.com/quickcalcs/index.cfm) or SOCR (Statistics Online Computational Resource; http://www.socr.ucla.edu/). A *p* value that was less than 0.05 was considered statistically significant.

## Supporting Information

Figure S1 Long-term (5 day) expression of dnTRF2 resulted in complex chromosomal fusions that were identified by M-FISH. (A) Examples of chromatid and chromosome fusions involving non-acrocentric chromosomes. Gray scale images of reverse-DAPI stained chromosomes are shown beside color panels. (B) An example of a complex fusion involving 7 chromosomes. Gray scale image to the right of the color panel shows the reverse-DAPI stained chromosomes. The color key/karyogram for the M-FISH experiments is shown in the right panel. Chromosomes are listed from HSA1 (*Homo sapiens* chromosome 1) (top left) to the HSAY (bottom right). (C) In 5-day inductions, acrocentric (13, 14, 15, 21, 22) fusions predominated (n = 1156) over acrocentric-non-acrocentric fusions. Each chromosome in the human karyotype is listed along the X-axis with the acrocentric chromosomes highlighted in **bold**. The acrocentrics alone are plotted along the Z-axis, and the number of fusions is plotted on the Y-axis.
(0.77 MB TIF)

Figure S2 Incidence and molecular structure of specific iROBs. The incidence of specific acrocentric fusions was determined from metaphase FISH data in two independent subclones of HTC75 dnTRF2-expressing cells. (A) In line T19, each type of iROB was observed, but certain ones, such as rob(13;13) occurred more frequently and sooner. Other iROBs, particularly those involving small acrocentrics HSA21 and HSA22, were predominantly formed after longer-term dnTRF2 expression. Asterisks indicate statistical differences in the proportion of each iROBs observed at 36 hours versus 5 days (*p*<0.05). “n” represents the total number of acrocentric fusions. (B) In line T4, almost every type of iROB occurred. Some, like irob(14;22) occurred early, but others, like irob(14;21) were formed later (5 days). Other ROBs were formed both after short-term and persistent dnTRF2 expression. Asterisk denotes statistical difference in the frequency of a particular fusion at 36 hours versus 5 days (*p*<0.05). “n” represents the total number of acrocentric fusions. (C) The iROBs in clone T19 showed variable structure, even among the same type of iROB. Many iROBs were missing one or more acrocentric repeats. (D) The number of TEL FISH signals at the fusion sites of induced dicentric varied. Telomeric DNA was visualized using a PNA-telomere (C_2_TA_3_)_3_ probe that was biotin labeled and detected with Alexa Fluor 488-streptavidin.
(0.21 MB TIF)

Figure S3 Non-random radial positions of human chromosomes in HTC75 T19 (parental uninduced line). After standard 2D FISH, at least 50 nuclei per chromosome painting probe were subjected to erosion analysis as described in Text S1. The normalized chromosomal signal (mean [% probe signal/% DAPI signal]) within five concentric shells was plotted as a histogram. Shell 1 represents the nuclear edge whereas shell 5 represents the nuclear interior. Histograms are arranged by chromosome going from peripheral location (top left) to interior location (bottom right).
(0.16 MB TIF)

Figure S4 Markers of DNA damage are present at telomeres and acrocentric short arms sequences when telomeres are un-protected. (A) Immunostaining for γH2AX (green) combined with FISH with a PNA-telomere probe (red) showed that dnTRF2 expression correlated with increased DNA damage at telomeres. (B) Immunostaining for γH2AX followed by FISH with acrocentric short arm probes revealed that DNA damage also occurs at short arm sequences in dnTRF2 cells. A β-satellite probe was used for FISH (red) to show co-localization with γH2AX foci (green). (C) Immunostaining for γH2AX (green) followed by FISH with a control X centromere α-satellite probe (red) revealed little co-localization of the probe with damage before or after dnTRF2 expression. (D) Dot plots showing co-localization of γH2AX foci with specific DNA sequences, including telomeres, β-satellite DNA (β-sat) and 3 control regions. 6BAC1 and 6BAC2 are BAC probes specific for 2 different regions of human chromosome 6. Xcen represents the α-satellite region on human chromosome X. Neither euchromatic or repetitive DNA controls showed co-localization with γH2AX foci after dnTRF2 induction, while telomeric DNA and acrocentric β-satellite DNA showed significantly increased associations with DNA damage foci. *N.S.* = *not significant*. Scale bars = 5 µm. For each experiment, more than 22 nuclei were analyzed.
(2.48 MB TIF)

Figure S5 Dicentric behavior and centromere function over time. Centromere function of independent versions of iROBs was monitored by CENP-A immunostaining and centromeric FISH over 14 weeks. (A) This irob(13;14) remained functionally dicentric over 14 weeks, but it was present in both functionally dicentric and monocentric states during the experimental period. When functionally monocentric, CEN13 was always inactivated. (B) A second, independent irob(13;14) appeared to be functionally monocentric at the start of the timecourse, but switched to and remained functionally dicentric in most cells during the time. When functionally monocentric, CEN13 was inactivated, similar to the irob(13;14) in (A). (C) An irob(13;13) showed both functionally dicentric and monocentric states in different cells at the beginning of the timecourse, but the functionally monocentric class predominated at the end of 14 weeks. (D) An irob(14;14) that was functionally dicentric at t_6_ underwent centromere inactivation by t_12_. The number of functionally dicentric chromosomes decreased over time until the iROB was functionally monocentric in almost all cells at t_20_.
(0.11 MB TIF)

Figure S6 Strategy to quantitate and compare fluorescence signals at acrocentric centromeres before and after iROB formation and centromere inactivation. This method correlates the intensity of fluorescent DNA probes for specific acrocentric centromeres to the size of the α-satellite arrays. In uninduced cells, CEN14 and CEN21 were detected using fluorescent DNA probes free-lying chromosomes that were measured by segmenting fluorescence and counting the number of the pixels in each segment. In tetraploid HTC lines, the HSA21 homologues were distinctive. One pair had a large α-satellite array and bright FISH signal and was designated CEN21^L^. The other homologue pair had a small α-satellite array and was denoted as CEN21^S^. CEN14 signals appeared to be equivalent in size/fluorescence. However, one HSA14 (denoted by asterisk) was visually larger since it contained translocated material from another chromosome. If it was not involved in an iROB after dnTRF2 induction, this homologue was excluded from control measurements. After iROB formation, the CEN14 and CEN21 signals/pixel intensities on the ROB were segmented, measured, and reported as arbitrary fluorescence units (AFUs). It appeared from the metaphase analyses that one CEN21^S^ was involved in the irob(14;21), since two HSA21 with large CEN21 signals were still present as free-lying chromosomes. In this way, we could know which HSA21 was involved in the iROB. The range of AFUs were compared between free-lying and ROB centromeres and displayed as box plots. For HSA21, CEN21^ROB^ was compared to both CEN21^L^ and CEN21^S^, even though it was more likely that one CEN21^S^ homologue was involved in the iROB.
(1.50 MB TIF)

Figure S7 Model for a potential mechanism of centromere inactivation in dicentric human chromosomes. When a dicentric forms from two free-lying chromosomes, both centromeres are functional. However, one centromere may be inactivated by deletion of the portion of α-satellite DNA array that is associated with CENP-A, an epigenetic marker for centromere identity. Because the factors that load newly synthesized CENP-A into chromatin may require identification of “old” CENP-A or nearby chromatin marks, removal of the CENP-A core may no longer identify one α-satellite array as centromeric. Consequently new CENP-A and other kinetochore proteins would not be replenished at this centromere, moving it into an inactive state.
(1.45 MB TIF)

Table S1 Occurrence of specific chromosome fusions in 36-hour, 3-day and 5-day inductions of dnTRF2 in two HTC75 clone T4. Fusions are listed by chromosome, starting from HSA1 to HSAY. Numbers in parentheses represent the number of times a particular fusion was observed in the cell population for a specific time point.
(0.06 MB DOC)

Table S2 Occurrence of specific chromosome fusions in 36-hour, 3-day and 5-day inductions of dnTRF2 in two HTC75 clone T19. Fusions are listed by chromosome, starting from HSA1 to HSAY. Numbers in parentheses represent the number of times a particular fusion was observed in the cell population for a specific time point.
(0.06 MB DOC)

Table S3 Expected versus observed incidence of common chromosome fusions in dnTRF2 inducible dicentric assay. Chromosome position influences chromosomal interactions in cancer. Asterisks * denote recurrent chromosomal interactions that are commonly associated with cancer. The plus (+) denotes the most common non-Robertsonian translocation in humans.
(0.06 MB DOC)

Text S1 Erosion analysis of nuclei and chromosomal painting probes.
(0.06 MB PDF)
