# Supplementary figures and images for: Telomere Disruption Results in Non-Random Formation of De Novo Dicentric Chromosomes Involving Acrocentric Human Chromosomes
Source: PLoS Genet. 2010 Aug 12;6(8):e1001061. doi: 10.1371/journal.pgen.1001061 (PMC2920838; doi:10.1371/journal.pgen.1001061)

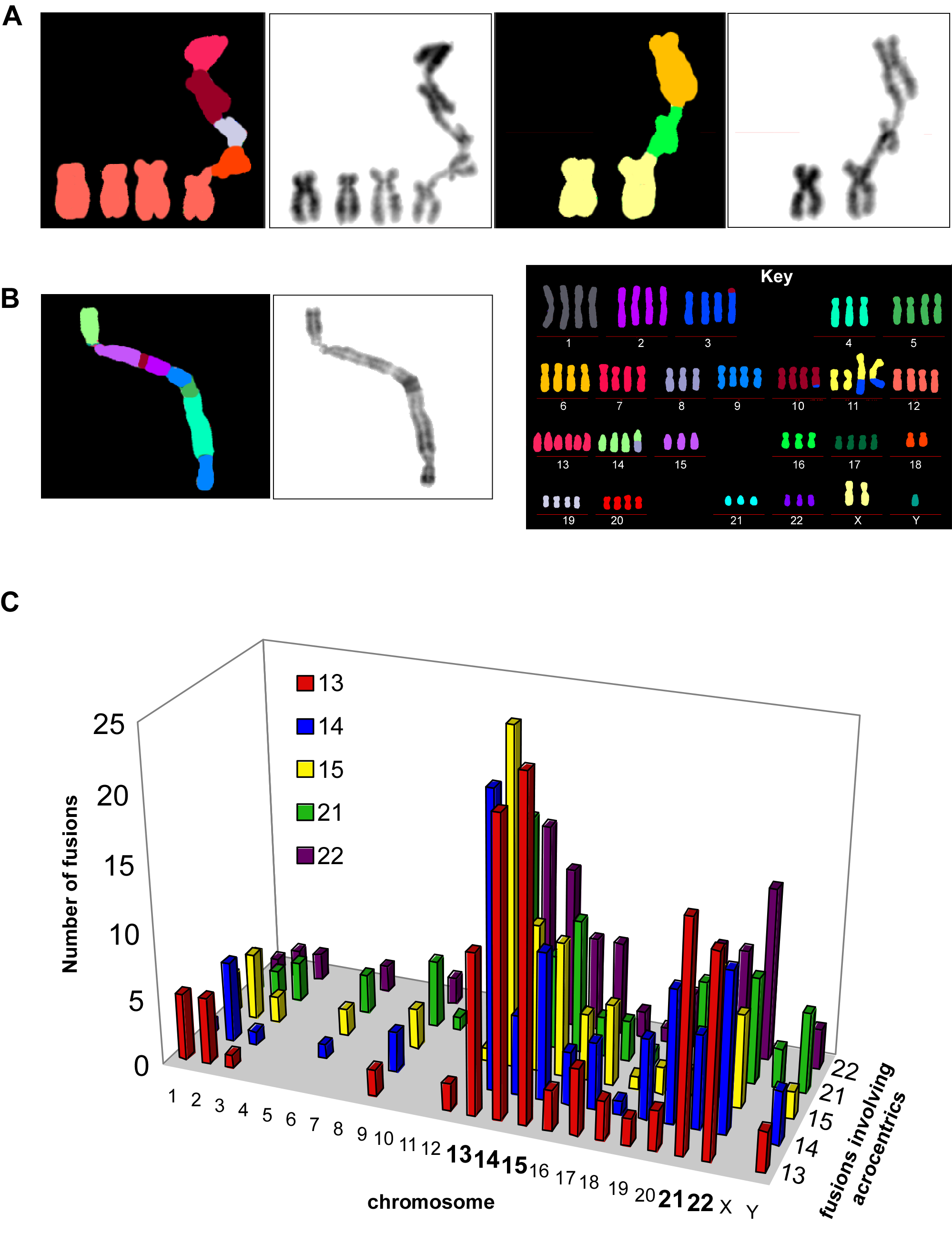

Supplement: Figure S1 — Long-term (5 day) expression of dnTRF2 resulted in complex chromosomal fusions that were identified by M-FISH. (A) Examples of chromatid and chromosome fusions involving non-acrocentric chromosomes. Gray scale images of reverse-DAPI stained chromosomes are shown beside color panels. (B) An example of a complex fusion involving 7 chromosomes. Gray scale image to the right of the color panel shows the reverse-DAPI stained chromosomes. The color key/karyogram for the M-FISH experiments is shown in the right panel. Chromosomes are listed from HSA1 (Homo sapiens chromosome 1) (top left) to the HSAY (bottom right). (C) In 5-day inductions, acrocentric (13, 14, 15, 21, 22) fusions predominated (n = 1156) over acrocentric-non-acrocentric fusions. Each chromosome in the human karyotype is listed along the X-axis with the acrocentric chromosomes highlighted in bold. The acrocentrics alone are plotted along the Z-axis, and the number of fusions is plotted on the Y-axis. (0.77 MB TIF) [file pgen.1001061.s001.tif]

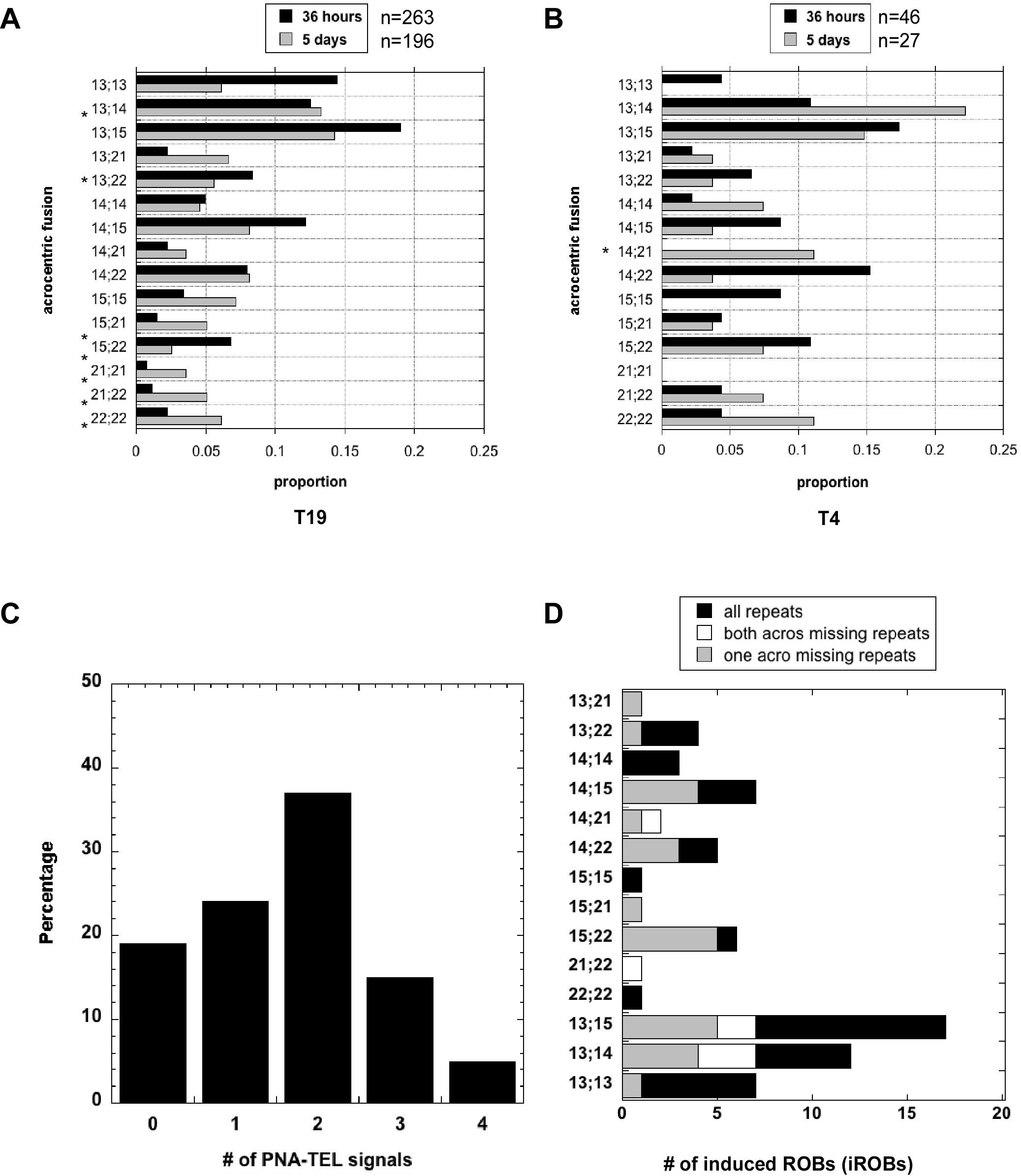

Supplement: Figure S2 — Incidence and molecular structure of specific iROBs. The incidence of specific acrocentric fusions was determined from metaphase FISH data in two independent subclones of HTC75 dnTRF2-expressing cells. (A) In line T19, each type of iROB was observed, but certain ones, such as rob(13;13) occurred more frequently and sooner. Other iROBs, particularly those involving small acrocentrics HSA21 and HSA22, were predominantly formed after longer-term dnTRF2 expression. Asterisks indicate statistical differences in the proportion of each iROBs observed at 36 hours versus 5 days (p<0.05). “n” represents the total number of acrocentric fusions. (B) In line T4, almost every type of iROB occurred. Some, like irob(14;22) occurred early, but others, like irob(14;21) were formed later (5 days). Other ROBs were formed both after short-term and persistent dnTRF2 expression. Asterisk denotes statistical difference in the frequency of a particular fusion at 36 hours versus 5 days (p<0.05). “n” represents the total number of acrocentric fusions. (C) The iROBs in clone T19 showed variable structure, even among the same type of iROB. Many iROBs were missing one or more acrocentric repeats. (D) The number of TEL FISH signals at the fusion sites of induced dicentric varied. Telomeric DNA was visualized using a PNA-telomere (C2TA3)3 probe that was biotin labeled and detected with Alexa Fluor 488-streptavidin. (0.21 MB TIF) [file pgen.1001061.s002.tif]

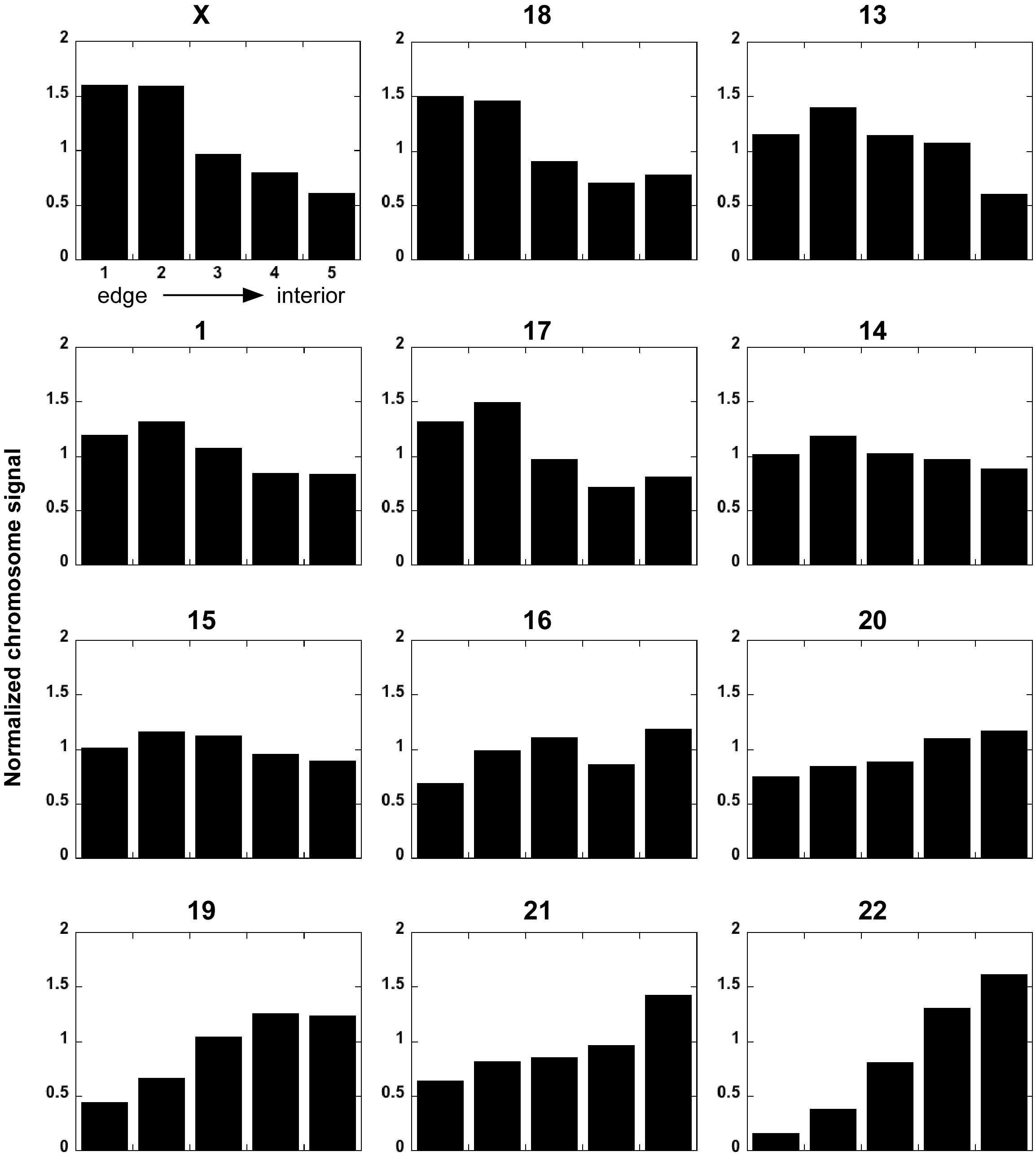

Supplement: Figure S3 — Non-random radial positions of human chromosomes in HTC75 T19 (parental uninduced line). After standard 2D FISH, at least 50 nuclei per chromosome painting probe were subjected to erosion analysis as described in Text S1. The normalized chromosomal signal (mean [% probe signal/% DAPI signal]) within five concentric shells was plotted as a histogram. Shell 1 represents the nuclear edge whereas shell 5 represents the nuclear interior. Histograms are arranged by chromosome going from peripheral location (top left) to interior location (bottom right). (0.16 MB TIF) [file pgen.1001061.s003.tif]

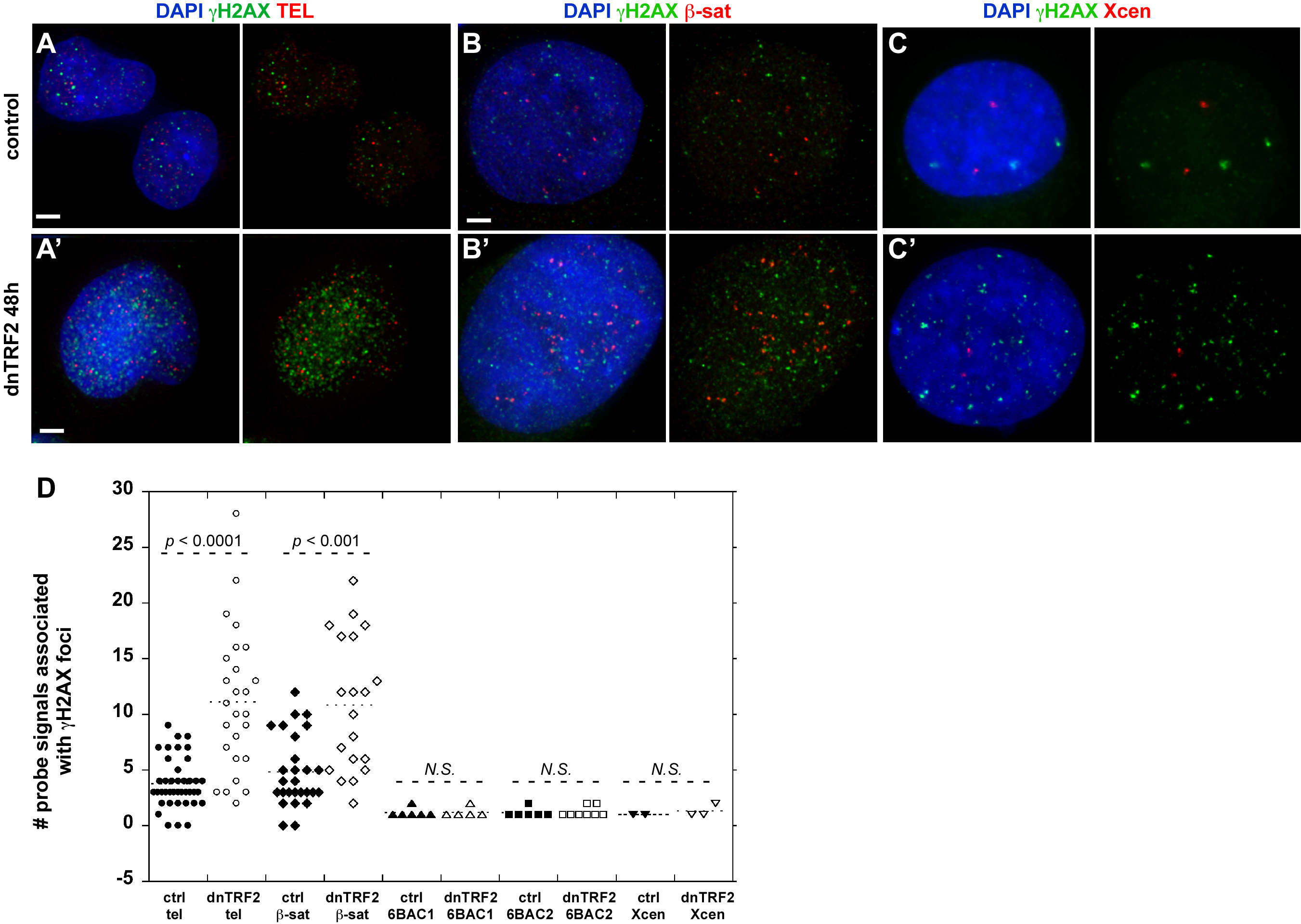

Supplement: Figure S4 — Markers of DNA damage are present at telomeres and acrocentric short arms sequences when telomeres are un-protected. (A) Immunostaining for γH2AX (green) combined with FISH with a PNA-telomere probe (red) showed that dnTRF2 expression correlated with increased DNA damage at telomeres. (B) Immunostaining for γH2AX followed by FISH with acrocentric short arm probes revealed that DNA damage also occurs at short arm sequences in dnTRF2 cells. A β-satellite probe was used for FISH (red) to show co-localization with γH2AX foci (green). (C) Immunostaining for γH2AX (green) followed by FISH with a control X centromere α-satellite probe (red) revealed little co-localization of the probe with damage before or after dnTRF2 expression. (D) Dot plots showing co-localization of γH2AX foci with specific DNA sequences, including telomeres, β-satellite DNA (β-sat) and 3 control regions. 6BAC1 and 6BAC2 are BAC probes specific for 2 different regions of human chromosome 6. Xcen represents the α-satellite region on human chromosome X. Neither euchromatic or repetitive DNA controls showed co-localization with γH2AX foci after dnTRF2 induction, while telomeric DNA and acrocentric β-satellite DNA showed significantly increased associations with DNA damage foci. N.S. = not significant. Scale bars = 5 µm. For each experiment, more than 22 nuclei were analyzed. (2.48 MB TIF) [file pgen.1001061.s004.tif]

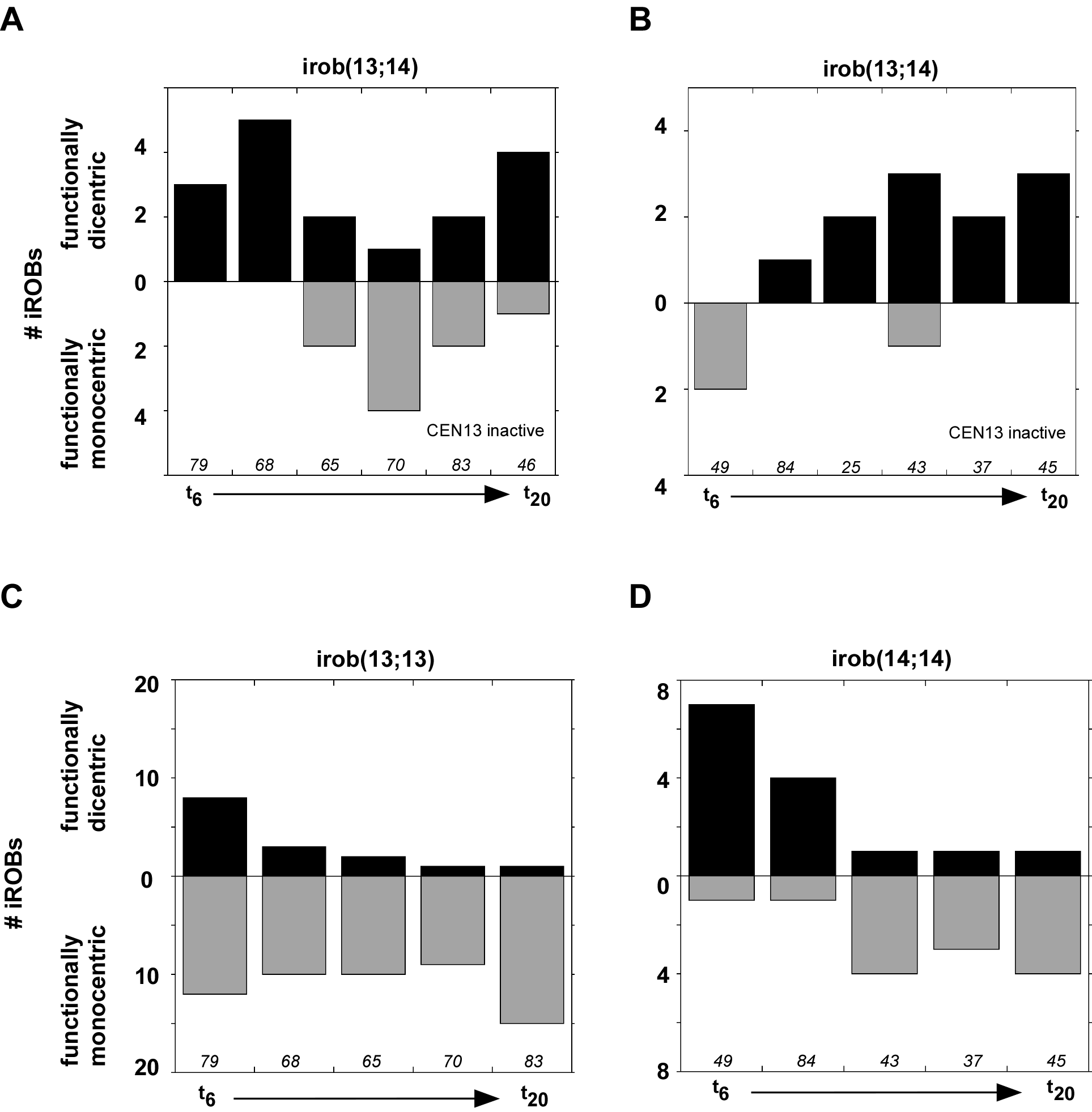

Supplement: Figure S5 — Dicentric behavior and centromere function over time. Centromere function of independent versions of iROBs was monitored by CENP-A immunostaining and centromeric FISH over 14 weeks. (A) This irob(13;14) remained functionally dicentric over 14 weeks, but it was present in both functionally dicentric and monocentric states during the experimental period. When functionally monocentric, CEN13 was always inactivated. (B) A second, independent irob(13;14) appeared to be functionally monocentric at the start of the timecourse, but switched to and remained functionally dicentric in most cells during the time. When functionally monocentric, CEN13 was inactivated, similar to the irob(13;14) in (A). (C) An irob(13;13) showed both functionally dicentric and monocentric states in different cells at the beginning of the timecourse, but the functionally monocentric class predominated at the end of 14 weeks. (D) An irob(14;14) that was functionally dicentric at t6 underwent centromere inactivation by t12. The number of functionally dicentric chromosomes decreased over time until the iROB was functionally monocentric in almost all cells at t20. (0.11 MB TIF) [file pgen.1001061.s005.tif]

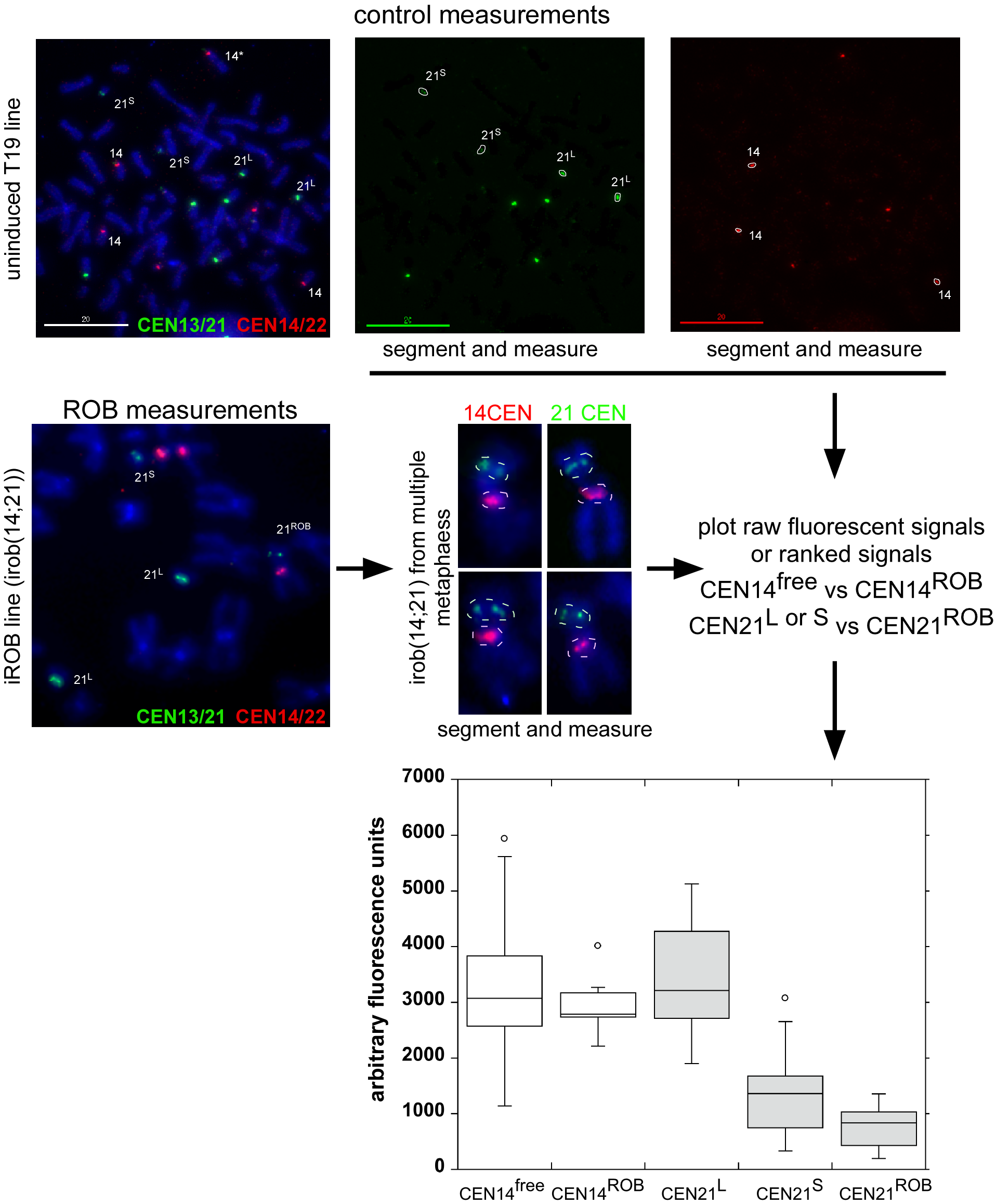

Supplement: Figure S6 — Strategy to quantitate and compare fluorescence signals at acrocentric centromeres before and after iROB formation and centromere inactivation. This method correlates the intensity of fluorescent DNA probes for specific acrocentric centromeres to the size of the α-satellite arrays. In uninduced cells, CEN14 and CEN21 were detected using fluorescent DNA probes free-lying chromosomes that were measured by segmenting fluorescence and counting the number of the pixels in each segment. In tetraploid HTC lines, the HSA21 homologues were distinctive. One pair had a large α-satellite array and bright FISH signal and was designated CEN21L. The other homologue pair had a small α-satellite array and was denoted as CEN21S. CEN14 signals appeared to be equivalent in size/fluorescence. However, one HSA14 (denoted by asterisk) was visually larger since it contained translocated material from another chromosome. If it was not involved in an iROB after dnTRF2 induction, this homologue was excluded from control measurements. After iROB formation, the CEN14 and CEN21 signals/pixel intensities on the ROB were segmented, measured, and reported as arbitrary fluorescence units (AFUs). It appeared from the metaphase analyses that one CEN21S was involved in the irob(14;21), since two HSA21 with large CEN21 signals were still present as free-lying chromosomes. In this way, we could know which HSA21 was involved in the iROB. The range of AFUs were compared between free-lying and ROB centromeres and displayed as box plots. For HSA21, CEN21ROB was compared to both CEN21L and CEN21S, even though it was more likely that one CEN21S homologue was involved in the iROB. (1.50 MB TIF) [file pgen.1001061.s006.tif]

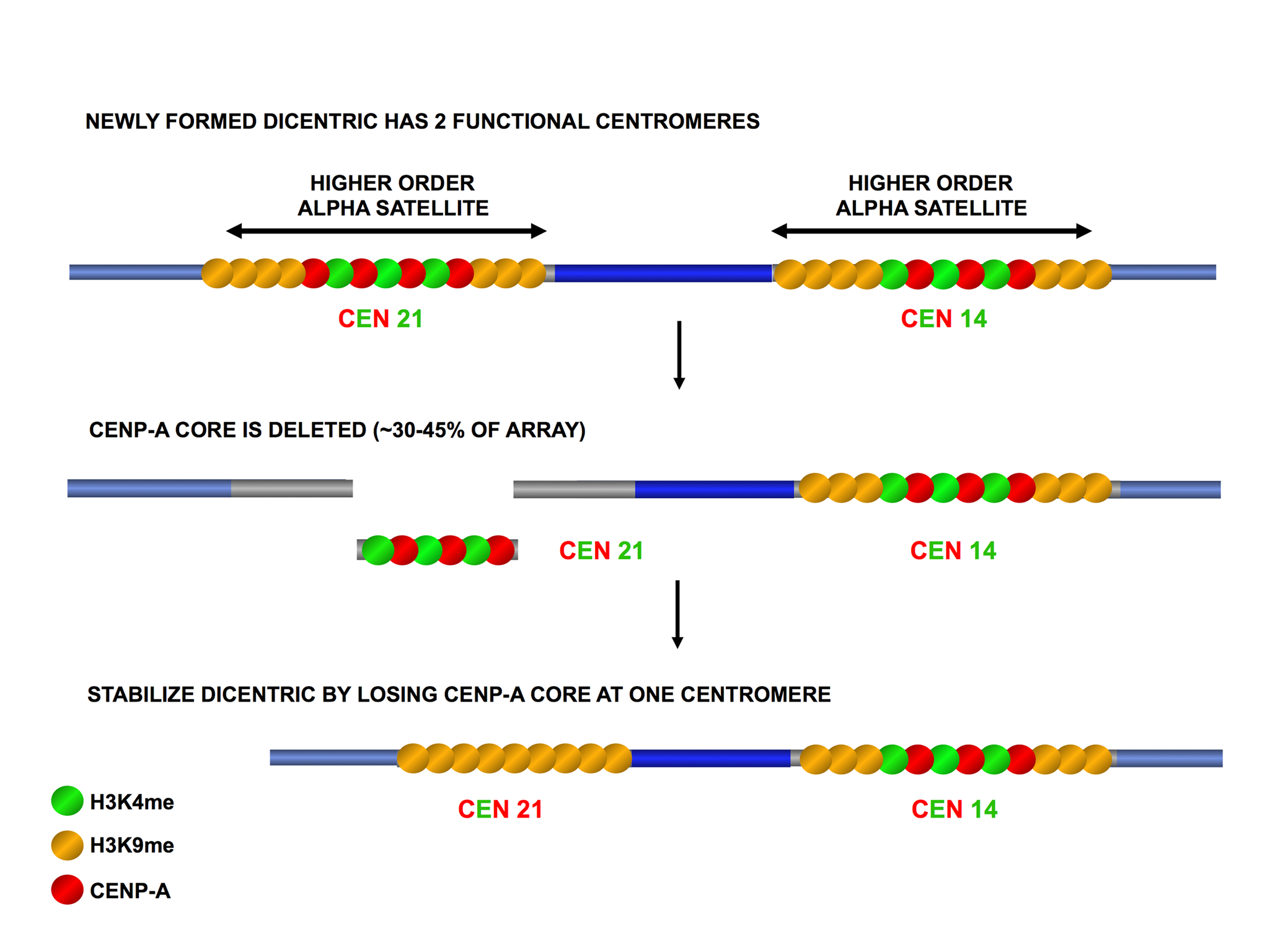

Supplement: Figure S7 — Model for a potential mechanism of centromere inactivation in dicentric human chromosomes. When a dicentric forms from two free-lying chromosomes, both centromeres are functional. However, one centromere may be inactivated by deletion of the portion of α-satellite DNA array that is associated with CENP-A, an epigenetic marker for centromere identity. Because the factors that load newly synthesized CENP-A into chromatin may require identification of “old” CENP-A or nearby chromatin marks, removal of the CENP-A core may no longer identify one α-satellite array as centromeric. Consequently new CENP-A and other kinetochore proteins would not be replenished at this centromere, moving it into an inactive state. (1.45 MB TIF) [file pgen.1001061.s007.tif]
