# Supplementary material for: Telomere Disruption Results in Non-Random Formation of De Novo Dicentric Chromosomes Involving Acrocentric Human Chromosomes
Source: PLoS Genet. 2010 Aug 12;6(8):e1001061. doi: 10.1371/journal.pgen.1001061 (PMC2920838; doi:10.1371/journal.pgen.1001061)
Supplement: Table S1 — Occurrence of specific chromosome fusions in 36-hour, 3-day and 5-day inductions of dnTRF2 in two HTC75 clone T4. Fusions are listed by chromosome, starting from HSA1 to HSAY. Numbers in parentheses represent the number of times a particular fusion was observed in the cell population for a specific time point. (0.06 MB DOC) [file pgen.1001061.s008.doc]

**Table S1 Identity of dnTRF2-induced chromosome fusions in line T4**

| **Chromosome** | **Fusions 36h** | **Fusions 3d** | **Fusions 5d** |
| --- | --- | --- | --- |
| **HSA1** |  | 1ptid;2qtid  1ptid;3qtid  1ptid;9qtid  1qtid;8qtid  1qtid;14ptid  1p;7p  1p;12p  1p;12q | 1ptid;Yptid  1ptid;14qtid  1ptid;17qtid  1p;20q  1;20 |
| **HSA2** | 2;11 (2) | 2qtid;5tid  2ptid;7qtid  2qtid;20ptid  2q;17q (2) | 2q;15p  2;20 |
| **HSA3** | 3;14 | 3;6ptid  3tid;8ptid  3tid;10q  3qtid;20ptid  3;10p |  |
| **HSA4** | del(4);12 | 4ptid;14qtid  4ptid;16qtid  4qtid;7ptid  4p;19p | 4;13  4;16 |
| **HSA5** |  | 5qtid;11ptid  5tid;13q | 5ptid;5ptid  5qtid;der8;14q;8  5ptid;16qtid  5;10 |
| **HSA6** |  |  | 6ptid;13qtid  6qtid;17ptid  6p;12p  6;13 (3)  6;16q  6;19 |
| **HSA7** |  | 7qtid;12qtid  7qtid;21qtid | 7ptid;15ptid  7qtid;16qtid  7ptid;17ptid  7;8  7q;8q |
| **HSA8** |  | 8ptid;11qtid  8ptid;12ptid  8qtid;10qtid | 8qtid;9qtid  8p;10p |
| **HSA9** | 9;14 | 9qtid;14qtid  9qtid;19ptid  9qtid;22qtid | 9;9  9q;der11  9;20 |
| **HSA10** | 10ptid;21ptid | 10ptid;11qtid  der(10);der(11)  10q;14q  10q;19p  10ptid;22ptid | 10ptid;13qtid  10qtid;19qtid  10;12p  10;13p  10;20  10p;21  10p;22p |
| **HSA11** | 11ptid;19ptid | 11ptid;12qtid  11ptid;16qtid  11qtid;20qtid | 11ptid;17ptid  11qtid;20qtid  11q;16  der11p;17p |
| **HSA12** | 12;Y | 12p;20p | 12ptid;18ptid  12qtid;20ptid  12qtid;21ptid  12;17 (2)  12q;22  12q;Yq |
| **HSA13** | 13ptid;13ptid (2)  13ptid;14ptid  13ptid;15ptid  13p;19qtid  13ptid;21ptid  13ptid;22ptid  13p;14p (4)  13p;15p (7)  13p;22p  13p;22p | 13ptid;14ptid  13ptid;15ptid (3)  13ptid;22qtid  13p;14p (5)  13p;15p (2)  13p;14p (3)  13p;21q  13q;21p  13p;22p (3) | 13ptid;15ptid  13ptid;19ptid  13p;14p (6)  13p;15p (3)  13p;20q  13p;21p  13p;22p  13;Xq |
| **HSA14** | 14ptid;14ptid  14ptid;15ptid (3)  14ptid;20ptid (2)  14ptid;22ptid (4)  14p;15p  14p;22p (3) | 14ptid;14qtid  14ptid;15ptid  14ptid;17qtid  14qtid;17qtid  14ptid;22ptid (2)  14ptid;Xqtid  14p;14p (2)  14p;21p  14p;22p (3)  14;Y | 14ptid;21ptid  14ptid;22ptid  14;der10;3  14p;14p (2)  14p;15p  14p;17p  14p;21p (2)  14q;Yq |
| **HSA15** | 15ptid;15ptid  15ptid;22ptid (2)  15p;15p (3)  15p;21p (2)  15p;22p (3) | 15ptid;22ptid  15qtid;derXtid  15p;15p (3)  15p;21p  15p;22p (2) | 15ptid;21ptid  15ptid;22ptid  15;17  15p;22p  15q;Xp |
| **HSA16** |  |  | 16qtid;19ptid  16q;20q (2) |
| **HSA17** |  | 17ptid;21ptid  17ptid;22qtid  17q;22q |  |
| **HSA18** |  |  | 18qtid;19ptid |
| **HSA19** |  | 19qtid;19qtid  19ptid;22qtid  19;21 | 19qtid;20ptid  19qtid;21qtid |
| **HSA20** | 20ptid;22ptid | 20ptid;20qtid | 20q;21p |
| **HSA21** | 21p;22p (2) | 21qtid;21qtid | 21p;21p  21p;22q |
| **HSA22** | 22ptid;22ptid  22p;22p | 22p;22p | 22ptid;22ptid  22p;22p (2)  22p;Yp |
| **HSAX** |  | Xq;9p  ring (X) | Xptid;17qtid  Xqtid;19ptid |
| **HSAY** |  | Yp;Yp |  |
