# Supplementary material for: Telomere Disruption Results in Non-Random Formation of De Novo Dicentric Chromosomes Involving Acrocentric Human Chromosomes
Source: PLoS Genet. 2010 Aug 12;6(8):e1001061. doi: 10.1371/journal.pgen.1001061 (PMC2920838; doi:10.1371/journal.pgen.1001061)
Supplement: Table S2 — Occurrence of specific chromosome fusions in 36-hour, 3-day and 5-day inductions of dnTRF2 in two HTC75 clone T19. Fusions are listed by chromosome, starting from HSA1 to HSAY. Numbers in parentheses represent the number of times a particular fusion was observed in the cell population for a specific time point. (0.06 MB DOC) [file pgen.1001061.s009.doc]

**Table S2 Identity of dnTRF2-induced chromosome fusions in line T19**

| **Chromosome** | **Fusions 36h** | **Fusions 3d** | **Fusions 5d** |
| --- | --- | --- | --- |
| **HSA1** | 1;7 | 1qtid;1qtid  1;5  1ptid;22qtid | 1ptid;1ptid (4)  1ptid;1qtid  1qtid;1qtid  1p;1p (3)  1p;1q (2)  ring(1) (2)  1;2  1;3 (11)  1;4 (4)  1q;5p  1;6 (2)  1;8 (4)  1;9 (18)  1;11 (8)  1;12 (14)  1;13 (6)  1;14 (2)  1;15 (3)  1;16 (8)  1;17 (15)  1;18 (8)  1;19  1;20 (3)  1;21 (2)  1;22  X;1 (3)  Y;1 (9) |
| **HSA2** |  | 2qtid;7ptid  2ptid;11qtid  2;9  2;17p | 2;3  2q;5p (2)  2;6 (4)  2;8  2;9 (8)  2;9  2;11 (2)  2;12 (16)  2;13 (5)  2;14 (8)  2;15 (6)  2;16 (2)  2;17 (9)  2;18 (5)  2;19 (3)  2;21 (2)  2;22 (4)  X;2 (7)  Y;2 |
| **HSA3** |  | 3;6ptid  3;7q (4) | 3ptid;3ptid (2)  3p;3q  3ptid;3qtid  3qtid;3qtid  3;4 (4)  3p;5p  3q;5p  3;6  3;7  3;8 (2)  3;9 (11)  3;11 (4)  3;12 (21)  3;13  3;14  3;15 (2)  3;16 (16)  3;17 (11)  3;18 (7)  3;19 (3)  3;20 (2)  3;21 (3)  3;22 (2)  3;X (2)  3;Y (4) |
| **HSA4** | 4ptid;21p |  | 4ptid;4ptid  4ptid;4qtid (2)  4p;4p (2)  4;5 (3)  4;9 (6)  4;16 (3) |
| **HSA5** | 5;13 | 5qtid;12qtid  5q;14q  der5qtid;der16tid  5qtid;18 | 5ptid;5ptid  5qtid;5qtid  5ptid;5qtid  5;16q (4)  5;16p  5;16 (4) |
| **HSA6** | 6p;20q | 6ptid;11qtid  6qtid;14qtid | 6ptid;6qtid (3)  6ptid;6ptid (2)  6qtid;6qtid (2)  6q;6q  6;7 (8)  6;9 (6)  6;14  6;15 (2)  6;17 (5)  6;19 (4)  6;20 (2)  6;21 (3)  6;22 (4)  6;Y (5) |
| **HSA7** |  | 7ptid;7qtid self (chromatid ring)  7;8  7;10  7qtid;14qtid  7qtid;15ptid  7ptid;22qtid | 7;12 (2)  7;X |
| **HSA8** |  | 8p;Xp | 8qtid;8qtid  8;9 (2)  8;12 (3) |
| **HSA9** |  | 9;der11  9ptid;12qtid  9q;17q | 9ptid;9ptid (7)  9ptid;9qtid (6)  9qtid;9qtid (2)  9q;9q  9;11 (4)  9;12 (5)  9;13 (3)  9;14 (2)  9;15 (4)  9;16 (16)  9;17 (21)  9;18 (22)  9;19 (5)  9;20 (2)  9;21 (9)  9;22 (3)  9;X (6)  9;Y (8) |
| **HSA10** | 10ptid;17ptid | 10qtid;12qtid  10ptid;18ptid  10q;19q | 10;17  10;19  10;20  10;21  10;X (3)  10;Y |
| **HSA11** | 11ptid;19ptid |  | 11ptid;11ptid  11ptid;11qtid  11qtid;11qtid (2)  11;12 (2)  11;16 (5)  11;17 (6)  11;18 (11)  11;19 (2)  11;Y (4) |
| **HSA12** |  | 12q;17q  12q;21p  12ptid;22qtid | 12ptid;12ptid (2)  12ptid;12qtid (2)  12qtid;12qtid (2)  12p;12p  12p;12q (2)  12q;12q (2)  12;12  ring(12) (2)  12;16 (6)  12;17 (10)  12;18 (5)  12;19 (2)  12;20 (2)  12;22 (3)  12;X (2)  12;Y (5) |
| **HSA13** | 13qtid ring (self) (2)  13p;13p (4)  13p;14p (3)  13p;15p (5)  13;18  13p;21p  13p;22p (5) | 13ptid;13ptid  13qtid;20qtid  13ptid;21ptid  13ptid;22ptid  13p;13p (3)  13q;13q  13p;14p (3)  13p;15p (2)  13p;21p  13p;22p (4) | 13ptid;13ptid (4)  13qtid;13qtid  13p;13p (3)  13p;13q  13q;13q  ring(13)  13p;14p (20)  13p;15p (24)  13;16 (3)  13;17 (7)  13;18 (6)  13p;21p (12)  13p;22p (9)  13;Y (4) |
| **HSA14** | 14p;14p (2)  14p;15p (5)  14p;21p (2)  14p;22p (3) | 14ptid;14ptid  14ptid;15ptid  14p;14p  14p;15p (4)  14p;21p  14qtid:Xptid  14;Y | 14ptid;14ptid (6)  14qtid;14qtid  14p;14p  14p;15p (11)  14;16 (7)  14;17 (8)  14;18 (2)  14;19 (2)  14;20 (2)  14p;21p (7)  14p;22p (15)  14;Y (4) |
| **HSA15** | 15ptid;15ptid  15ptid;18ptid (2)  15ptid;21ptid  15p;15p  15;16  15;17  15p;21p  15p;22p (2) | 15ptid;22ptid  15qtid;derXtid  15p;15p (3)  15p;21p  15p;22p (2) | 15ptid;15ptid (4)  15ptid;15qtid  15qtid;15qtid (2)  15p;15p (3)  15;16 (4)  15;17 (6)  15;18 (2)  15p;21p (10)  15p;22p (4)  15;Y (2) |
| **HSA16** |  | 16;21 | 16;16  16ptid;16ptid (5)  16ptid;16qtid (3)  16qtid;16qtid (5)  16p;16p (2)  16p;16q (2)  16q;16q (2)  16;17 (37)  16;18 (18)  16;19 (4)  16;20 (7)  16;21 (4)  16;22 (10)  16;Y (5) |
| **HSA17** |  | 17q;21q | 17;17 (3)  17ptid;17ptid  17qtid;17qtid (3)  17ptid;17qtid (5)  17p;17p (2)  17p;17q (7)  17q;17q (3)  ring(17)  17;18 (20)  17;19 (3)  17;20 (12)  17;21  17;22 (2)  17;X (2)  17;Y (7) |
| **HSA18** |  |  | 18;18 (3)  18ptid;18ptid  18ptid;18qtid (5)  18qtid;18qtid (3)  18p;18p (5)  18p;18q  18q;18q (3)  18;19 (8)  18;20 (9)  18;Y (8) |
| **HSA19** | 19qtid;20qtid | 19p;20q | 19;19  19ptid;19ptid (4)  19qtid;19qtid (2)  19p;19p (4)  19q;19q (3)  ring(19)  19;20 (5)  19;X (7)  19;Y (4) |
| **HSA20** | 20ptid;22ptid |  | 20;20  20ptid;20ptid (2)  20ptid;20qtid  20qtid;20qtid  20q;20q (2)  20;21 (4)  20;22 (6)  20;X (8)  20;Y |
| **HSA21** | 21ptid;21ptid | 21ptid;21ptid | 21ptid;21ptid (3)  21ptid;21qtid  21p;21p (2)  21q;21q  21p;22p (9)  21;X (5)  21;Y (7) |
| **HSA22** | 22ptid;22ptid  22p;22p | 22ptid;22ptid  22p;22p | 22ptid;22ptid (4)  22ptid;22qtid  22qtid;22qtid  22p;22p (4)  22q;22q (2)  22;Y |
| **HSAX** | Xptid;Yptid | Xptid;Yptid | Xptid;Xptid  Xptid;Xqtid (2)  Xp;Xp  X;Y (5) |
| **HSAY** |  |  | Yp;Yq (2)  Yq;Yq |
