# Supplementary material for: Telomere Disruption Results in Non-Random Formation of De Novo Dicentric Chromosomes Involving Acrocentric Human Chromosomes
Source: PLoS Genet. 2010 Aug 12;6(8):e1001061. doi: 10.1371/journal.pgen.1001061 (PMC2920838; doi:10.1371/journal.pgen.1001061)
Supplement: Table S3 — Expected versus observed incidence of common chromosome fusions in dnTRF2 inducible dicentric assay. Chromosome position influences chromosomal interactions in cancer. Asterisks * denote recurrent chromosomal interactions that are commonly associated with cancer. The plus (+) denotes the most common non-Robertsonian translocation in humans. (0.06 MB DOC) [file pgen.1001061.s010.doc]

**Table S3 Frequencies of non-acrocentric fusions in dnTRF2 inducible assay.**

| Fusion | Expected | Observed | **χ2 value** | Df | P | Significance |
| --- | --- | --- | --- | --- | --- | --- |
| 1;9 | 16/3321 | 18/1360 | 8.35 | 1 | 0.0039 | VS |
| 1;17 | 16/3321 | 15/1360 | 4.754 | 1 | 0.0292 | S |
| 2;12 | 16/3321 | 16/1360 | 5.873 | 1 | 0.0154 | S |
| 2;17 | 16/3321 | 11/1360 | 1.274 | 1 | 0.2589 | NS |
| 3;12 | 16/3321 | 21/1360 | 12.564 | 1 | 0.0004 | VS |
| 3;16 | 16/3321 | 16/1360 | 5.873 | 1 | 0.0154 | S |
| 8;14* | 12/3321 | 1/1360 | 1.94 | 1 | 0.1636 | NS |
| 8;22* | 9/3321 | 0/1360 | 2.416 | 1 | 0.1201 | NS |
| 9;17 | 16/3321 | 21/1360 | 12.564 | 1 | 0.0004 | VS |
| 9;18 | 6/3321 | 22/1360 | 31.135 | 1 | <0.0001 | VS |
| 9;22* | 9/3321 | 3/1360 | 0.239 | 1 | 0.625 | NS |
| 11;22+ | 12/3321 | 1/1360 | 1.94 | 1 | 0.1636 | NS |
| 12;15* | 9/3321 | 0/1360 | 2.416 | 1 | 0.1201 | NS |
| 15;17* | 12/3321 | 7/1360 | 0.246 | 1 | 0.6198 | NS |
| 17;18 | 12/3321 | 20/1360 | 15.89 | 1 | <0.0001 | VS |
| 17;20 | 16/3321 | 12/1360 | 1.974 | 1 | 0.1601 | NS |
| 17;22* | 9/3321 | 2/1360 | 0.214 | 1 | 0.6436 | NS |
| 18;19 | 12/3321 | 8/1360 | 0.695 | 1 | 0.4044 | NS |
| 19;20 | 8/1360 | 8/1360 | 0.056 | 1 | 0.8122 | NS |
| X;22 | 6/3321 | 4/1360 | 0.172 | 1 | 0.678 | NS |

Df=degrees of freedom; S=significant; VS=very significant; NS=not significant. Significance was calculated using a x-square test using Graphpad Statistics program. * indicate recurrent lymphoma and leukemia-related chromosomal interactions; + denotes common non-Robertsonian chromosomal interactions in humans
