# Supplementary material for: Telomere Disruption Results in Non-Random Formation of De Novo Dicentric Chromosomes Involving Acrocentric Human Chromosomes
Source: PLoS Genet. 2010 Aug 12;6(8):e1001061. doi: 10.1371/journal.pgen.1001061 (PMC2920838; doi:10.1371/journal.pgen.1001061)
Supplement: Text S1 — Erosion analysis of nuclei and chromosomal painting probes. (0.06 MB PDF) [file pgen.1001061.s011.pdf]

Stimpson_Supplemental Data

Experimental Procedures

Erosion analysis of nuclei and chromosomal painting probes

Preparation of flattened interphase nuclei for FISH was performed as previously described [1,2]. After FISH with whole chromosome painting probes (see Experimental Procedures), all nuclei were examined under a 100X oil immersion objective and images of random nuclei were collected. At least 50 digital images were collected using Smart Capture 2 (Digital Scientific) controlled by a Sensys camera. Chromosome positioning within interphase nuclei was determined by analyzing each image in a custom bespoke erosion analysis script written in IP Lab Spectrum (kind gift of P. Perry and W. Bickmore, MRC HGU, Edinburgh). The script divides the nucleus into five shells of equal area eroded from the periphery (shell 1) to the interior (shell 5). The script permits measurement of the amount of fluorescent signal from the chromosome paint and DAPI in each of the shells. Normalized probe signal was calculated by dividing the percentage of probe signal in each shell by the percentage of the DAPI signal in the same shell. The data were then present as histograms that displayed whether a particular chromosome signal was predominantly locate near the nuclear edge, in nuclear periphery or showed an intermediate distribution (i.e. equivalent probe distribution across multiple shells).

Supplemental References

1. Bridger JM, Boyle S, Kill IR, Bickmore WA (2000) Re-modelling of nuclear architecture in quiescent and senescent human fibroblasts. Curr Biol 10: 149-152.

2. Croft JA, Bridger JM, Boyle S, Perry P, Teague P, et al. (1999) Differences in the localization and morphology of chromosomes in the human nucleus. J Cell Biol 145: 1119-1131.
